# Clinicians’ Attitudes and Perceptions on the Adoption of AI in Mental Health Care: Scoping Review

**DOI:** 10.2196/92370

**Published:** 2026-09-11

**Authors:** Carly Hudson, Thuy Linh Phan, Marcus Randall

**Affiliations:** 1 Faculty of Health Sciences and Medicine, Bond University, 14 University Drive, Robina, Queensland, 4226, Australia, 61 5595 3361; 2 Bond Business School, Bond University, Robina, Queensland, Australia; 3 University of Queensland Business School, The University of Queensland, St Lucia, Queensland, Australia

**Keywords:** mental health care, clinician attitudes, clinical decision support system, AI, scoping review, PRISMA, Preferred Reporting Items for Systematic Reviews and Meta-Analyses

## Abstract

**Background:**

AI is increasingly being integrated into health care workflows, with growing interest in AI systems for documentation, screening, triage, monitoring, and decision support. Mental health care is particularly complex for AI implementation as clinical care largely depends on therapeutic relationships, contextual factors, empathic communication, and interpretation of subtle nonverbal cues. Although AI may offer opportunities to improve efficiency and access to care, its adoption is likely to depend on clinicians’ trust, ethical acceptability, safety, confidentiality, and clarity around professional responsibility.

**Objective:**

This scoping review aims to synthesize current evidence on mental health clinicians’ attitudes, perceptions, and beliefs regarding the use of AI tools within mental health care, including the perceived benefits, risks, acceptable use cases, and conditions considered necessary for implementation.

**Methods:**

A scoping review was conducted in accordance with Joanna Briggs Institute (JBI) guidance and PRISMA-ScR (Preferred Reporting Items for Systematic Reviews and Meta-Analyses Extension for Scoping Reviews) guidelines. Six databases (CINAHL, Embase, PsycINFO, PubMed, Scopus, Web of Science) were searched on May 5, 2026, for studies published from 2020 onwards. Studies were eligible if they examined clinicians’ attitudes, perceptions, or beliefs regarding AI in mental health care. Studies were screened by 2 reviewers, first by title and abstract, and then by full text. Data extraction included publication year, country, methodology, participant occupation, AI type, outcome domains, and relevant findings.

**Results:**

Database searches retrieved 12,356 records. Following screening, 35 records were included. Overall, clinicians demonstrated cautious optimism toward AI, particularly when positioned as supplementing instead of replacing clinical expertise. Perceived benefits centered on reducing administrative burden, supporting documentation, synthesizing large volumes of information, and improving access to care, particularly between sessions. However, clinicians also reported limited AI literacy and prior use, and were concerned about privacy, confidentiality, governance, data ownership, clinical safety, unsafe or inaccurate outputs, overreliance, unclear accountability, professional role boundaries, and impacts on therapeutic relationships. Clinicians emphasized the need for education, clear guidelines and governance, role clarity, co-designed systems, human oversight, and real-world, ongoing evaluation.

**Conclusions:**

This review is innovative in shifting focus from the technical performance of AI tools to the perspectives of the clinicians who will be expected to use, interpret, explain, and remain accountable for them in mental health care. Unlike previous reviews, this review provides a clinician-centered understanding of AI adoption, highlighting that acceptability depends not only on what AI can do but also on whether it can be integrated safely, ethically, and in ways that preserve professional judgment and therapeutic relationships. These findings suggest that implementation should begin with lower-risk (eg, administrative), clinician-facing applications, be supported by education and governance, and be evaluated in mental health settings before wider adoption. These insights provide practical direction for responsible, clinician-centered implementation across mental health services.

## Introduction

The implementation of AI is rapidly growing within health care, with increasing movement from theoretical modeling on retrospective datasets to real-time usage [1,2]. The recent growth in uptake of AI tools, particularly since the release of publicly available generative AI software, has further accelerated how AI might be used to support clinical communication, documentation, and decision making [2].

Mental health represents an important but equally challenging context for AI implementation [3]. Mental health conditions are complex in nature, and developing treatment plans requires careful consideration of the patient’s subjective experience and personal narratives, while relying on the therapeutic alliance, empathic communication, and other contextual factors [4,5]. As a result, despite advances in AI, the use of AI in mental health care raises questions about professional responsibility, patient autonomy, and the preservation of human-centered care [6,7]. These concerns are especially salient in mental health care, where inappropriate advice, underestimated risk, or loss of relational trust may have serious consequences for patient safety and engagement in future therapy [8]. In addition, the sensitivity of mental health information leads to concerns for confidentiality, data security, and appropriate governance when implementing AI systems [9].

Clinicians’ perspectives are central to acceptable and safe implementation of AI in health care [10]. Regardless of technical performance or abilities of AI tools, real-world usage will depend upon whether clinicians trust them, understand their limitations, perceive them as clinically useful, and believe that they can be used without compromising patient care [10]. As gatekeepers to AI implementation, clinicians’ attitudes may therefore influence whether AI tools are adopted or resisted [3,11,12]. Understanding clinicians’ perspectives is therefore essential for determining not only whether AI is acceptable in mental health care, but also which aspects of care clinicians consider appropriate for AI support, and which should remain primarily human-led.

To date, several reviews have examined the use of AI in mental health care, although their aims and scopes differ from those of the current review. Existing syntheses have predominantly evaluated the technical and clinical applications of AI for diagnosis, prediction, monitoring, and intervention, or the effectiveness and user experience of conversational agents and other AI-enabled digital interventions [13-15]. For instance, Cruz-Gonzalez et al [13] reviewed 85 studies of AI applications for mental health diagnosis, monitoring, and intervention, with a primary focus on the methods and performance of the technologies. Reviews of conversational agents have similarly concentrated on their effectiveness in improving mental health outcomes and the factors influencing user experience [14,15]. Other reviews have examined stakeholder perspectives, but have addressed different populations, technologies, or contexts from those of the current review. Young et al [16] synthesized patient and general public attitudes toward clinical AI across health care, rather than the perspectives of mental health clinicians specifically. Henzler et al [17] reviewed health care professionals’ perceived facilitators and barriers to AI adoption across medical specialties, but only one of the 72 included studies related to psychiatry, limiting the review’s ability to examine issues specific to mental health care. Rebelo et al [18] examined how AI may affect the tasks performed by mental health care workers, rather than comprehensively synthesizing clinicians’ attitudes, acceptable use cases, concerns, and perceived implementation requirements.

Together, existing reviews demonstrate growing interest in AI within mental health care, but they do not provide a comprehensive synthesis of primary evidence examining how mental health clinicians perceive the expanding range of AI applications. In particular, there remains a need to integrate evidence across professional groups, study designs, and AI technologies, and to examine not only clinicians’ overall attitudes but also the uses they consider acceptable, the perceived risks and benefits, and the conditions they believe are necessary for safe implementation. Given that clinicians’ willingness, confidence, and capability are central to the adoption of AI in practice, understanding these perspectives is essential for informing the design, evaluation, and implementation of AI tools within mental health services [3,11,12,19]. This scoping review therefore aims to synthesize current evidence on clinicians’ attitudes, perceptions, and beliefs regarding the use of AI tools within mental health care.

## Methods

### Protocol and Registration

This scoping review was conducted in accordance with the recommendations of the Joanna Briggs Institute (JBI) [20], and was reported in accordance with the PRISMA-ScR (Preferred Reporting Items for Systematic Reviews and Meta-Analyses Extension for Scoping Reviews) guidelines (Checklist 1) [19]. A protocol for this scoping review was prospectively registered with the Open Science Framework [21].

### Selection Criteria

The selection criteria for this review were developed using the population, concept, and context framework. The population of interest was comprised of clinicians or health professionals working in mental health care, including psychiatrists, psychologists, mental health nurses, medical professionals, counselors, social workers, and other allied health staff. Studies containing mixed participant groups (eg, those reporting attitudes of clinicians and patients) were eligible when findings relating to clinicians or health professionals were reported separately and could be extracted.

The concept of interest was clinicians’ attitudes, perceptions, beliefs, knowledge, acceptability, readiness, concerns, or experiences of AI. Studies in which AI was examined broadly were eligible, including generative AI, machine learning, clinical decision support systems, conversational agents, documentation tools, screening or monitoring systems, and predictive applications. The context was mental health care, including psychiatric, psychological, counseling, psychotherapy, and other mental health service settings.

Studies were eligible for inclusion if they reported empirical data relating to clinicians’ attitudes, perceptions, or beliefs regarding the use of AI in mental health care. This included studies using surveys, interviews, focus groups, mixed methods designs, or other approaches that captured clinicians’ perspectives. Studies were included where AI was examined broadly, or where a specific type of AI or use case was examined.

Studies were excluded if they evaluated technical performance, diagnostic accuracy, or clinical effectiveness of an AI tool without reporting on clinicians’ attitudes, perceptions, or beliefs. Studies were also excluded if they focused exclusively on patients, students, caregivers, developers, or the general public, unless clinicians’ perspectives were separately reported. Reviews, conference abstracts and proceedings, errata, editorials, poster presentations, commentaries, non–peer-reviewed documents, and articles not available in English were also excluded. Studies published before January 2020 were excluded to capture the most recent evidence, reflecting the rapid development and increasing accessibility of AI technologies, particularly generative AI. Non–peer-reviewed sources were excluded as the review aimed to synthesize primary empirical evidence concerning clinicians’ perspectives. Studies not available in English were excluded, as resources for translation were not available. No restrictions were applied according to geographical location, clinician discipline, participant age group, or type of mental health service.

### Information Sources

The final search was conducted on May 5, 2026, and included 6 databases searched individually: CINAHL (EBSCO), Embase (Elsevier), PsycINFO (Ovid), PubMed (National Library of Medicine), Scopus (Elsevier), and Web of Science (Clarivate), using both MeSH terms or other database-specific subject headings, and free-text keywords. The search was limited from January 1, 2020, to the date of the search using database filters, in order to capture the latest AI-focused research in this rapidly developing area. No additional registries, websites, print sources, or search methods were used.

### Search Strategy

The search strategy was developed by the authors, was not based on any previously published searches, and was reported in line with the PRISMA-S (Preferred Reporting Items for Systematic Reviews and Meta-Analyses Literature Search Extension) guidelines (Checklist 2). The search strategy was developed considering each aspect of the population-concept-context, in consultation with a research librarian, and was piloted and subsequently refined by the authors. The search encompassed key terms relating to (1) clinicians; (2) AI; (3) attitudes, perceptions, or beliefs; and (4) mental health.

The preliminary search strategy was developed in PubMed and piloted to assess the relevance of retrieved records. It was then translated for the remaining databases, with database-specific subject headings and syntax adapted to the requirements of each platform. Searches were executed by 1 author (CH). The full reproducible search strategies for all databases searched are available in Multimedia Appendix 1.

### Screening and Selection Process

The results of the database searches were imported into EndNote (Clarivate), and then uploaded into Covidence, a systematic review management tool [22]. Articles were deduplicated by Covidence (Veritas Health Innovation), and then screened by 2 authors (CH and MR), first by title and abstract, then by full text.

Eligibility criteria were operationalized using a standardized screening form in Covidence available to both reviewers. Before formal screening, both reviewers independently assessed a pilot sample of titles and abstracts. Differences in the interpretation of the eligibility criteria were discussed, and the screening guidance was refined before the remaining records were assessed. Both reviewers independently screened each title and abstract. Records considered potentially eligible by either reviewer proceeded to full-text assessment. The reviewers then independently assessed each full-text article against the eligibility criteria and recorded a primary reason for exclusion for articles that were excluded. Conflicts were discussed between the raters until a consensus was reached. Interrater agreement was assessed using Cohen κ.

### Data Charting

A data extraction template was developed in Microsoft Excel by 1 author (CH), which was piloted and refined in consultation with a second author (MR). The form was piloted using a subset of included studies selected to represent different study designs and AI applications. Following the pilot, the reviewers discussed the relevance of each field, and the form was refined to ensure consistency and that all data relevant to the review questions were captured.

Data items were extracted by 2 authors (CH and MR), who cross-checked results for accuracy and consistency. Discrepancies were resolved through discussion and consensus, with reference to the original publication. The charting form was updated iteratively where new relevant concepts emerged, and previously charted studies were reviewed following any changes to ensure consistent application across the dataset. Where information was not reported or could not be determined from the publication, it was recorded as “not reported.”

### Data Items

Extracted data included authors, year of publication, country, study designs, data collection methods, participants’ occupation, type of AI examined, study domains or topics explored, and findings relevant to clinicians’ attitudes, perceptions, and beliefs. Data were also extracted, where reported, on perceived benefits, risks, use cases, implementation considerations, governance issues, needs for education, impacts on professional roles, impacts on patients, and overall sentiments toward AI. Professional groups were initially recorded using the terminology reported by each study and were subsequently grouped into broader categories. AI applications were classified according to their described function. In both cases, categories were not mutually exclusive, and studies could contribute to more than one professional or AI application category.

### Synthesis of Results

Study characteristics were summarized descriptively using counts and percentages for publication year, geographical location, study design, participant profession, and type of AI application examined. Findings relating to clinicians’ attitudes, perceptions, and beliefs were synthesized narratively. Extracted findings were initially organized according to the review objectives and recurring concepts identified across the included studies. Similar findings were compared and grouped into broader descriptive domains through discussion between 2 reviewers (CH and MR). The developing domain structure was applied across all included studies and refined iteratively until it represented the range of perspectives reported.

The studies’ key findings were synthesized into nine domains: (1) AI knowledge, familiarity, and prior use; (2) attitudes, perceptions, and emotional responses; (3) perceived benefits and opportunities; (4) perceived risks, harms, and ethical concerns; (5) potential uses for AI in mental health care; (6) implementation, integration, and adoption readiness; (7) impact of AI on professional roles and future practice; (8) impact on the therapeutic relationship; and (9) education, training, policy, and governance needs.

## Results

### Selection of Sources

**Figure 1.**
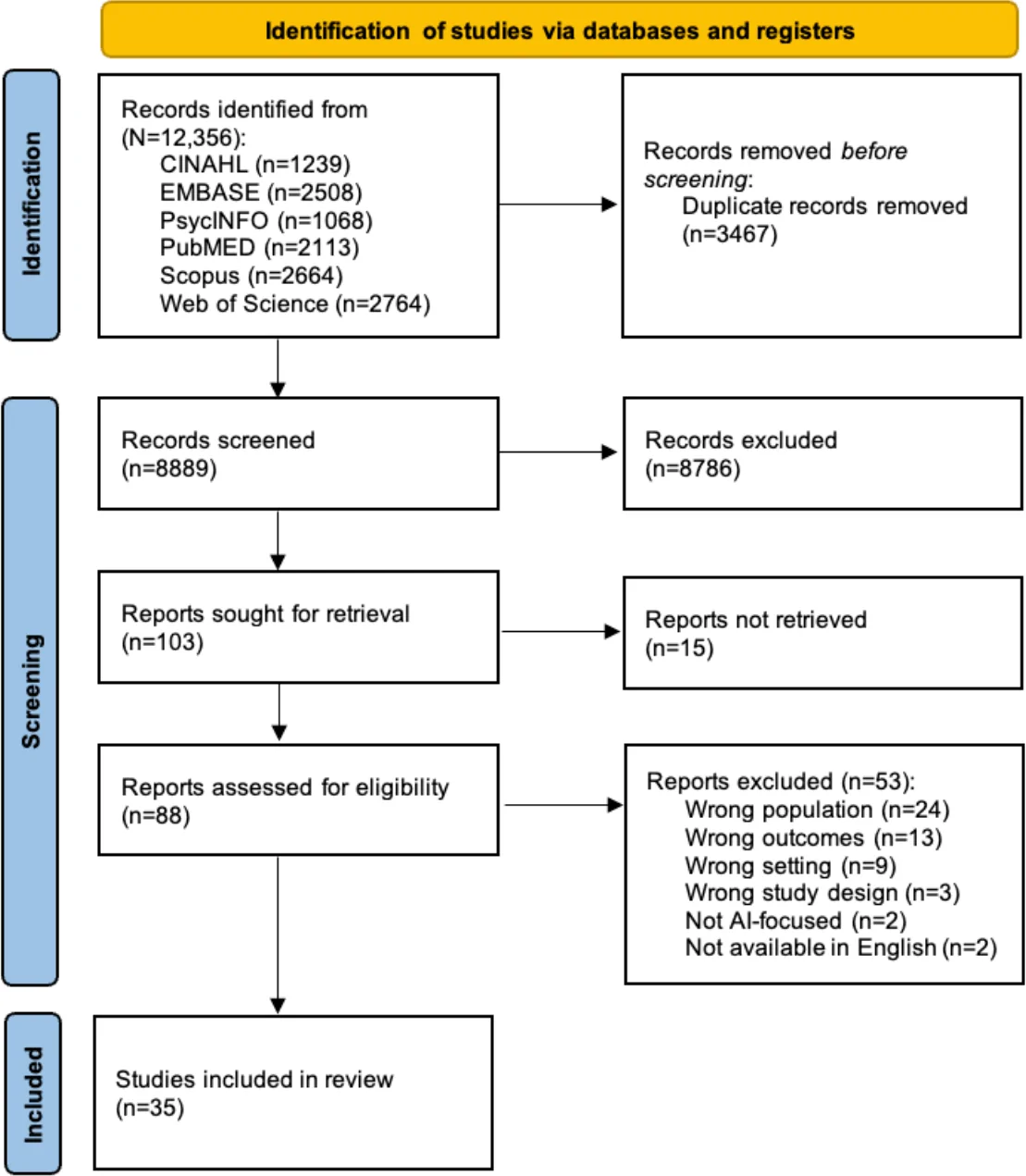
PRISMA (Preferred Reporting Items for Systematic Reviews and Meta-Analyses) flow diagram.

The database searches returned a total of 12,356 records. Following the removal of 3467 duplicates, 8889 sources were screened by title and abstract, with 8786 being deemed irrelevant. The remaining 103 articles were sought for retrieval, with 15 of these being excluded due to the full text being unavailable. The remaining 88 full texts were screened, with 53 excluded as they did not meet the inclusion criteria for the following reasons: wrong population (n=24), wrong outcomes (n=13), wrong setting (n=9), wrong study design or publication type (n=3), not being AI-focused (n=2), and studies not available in English (n=2). The raters demonstrated moderate agreement when screening (Cohen κ=0.60). A total of 35 articles were included in the final review (Figure 1) [3,11,12,23-54]

### Characteristics of Included Studies

**Figure 2.**
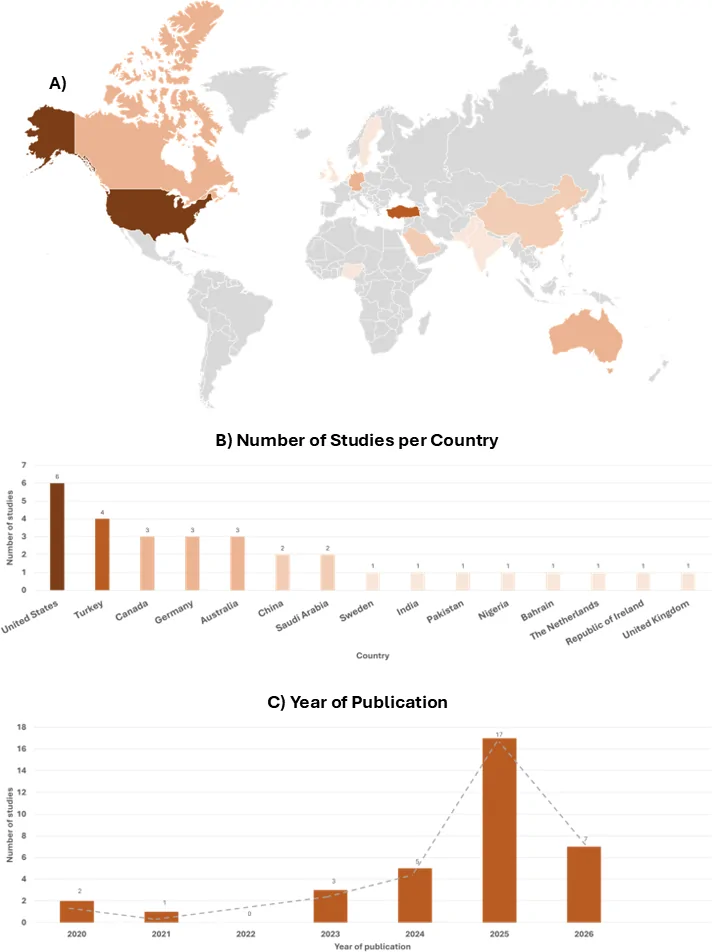
(A) World map displaying the study locations, (B) number of studies per country, and (C) year of publication.

The included studies were published between 2020 and 2026, with most (n=29, 82.9%) being published from 2024 onwards. Studies were conducted globally, targeting populations in North America (n=9, 25.7%) [30,35,36,41,46-48,51,54], the Middle East (n=7, 20.0%) [11,25-27,39,40,52], Europe (n=6, 17.1%) [28,34,44,45,49,53], Asia (n=4, 11.4%) [23,32,43,50], Australia (n=3, 8.6%) [3,12,37], and Africa (n=1, 2.9%) [24]. Four (11.4%) studies were conducted globally [29,33,38,42] and 1 study (2.9%) was conducted across both Germany and the United States [31] (Multimedia Appendix 2, Figure 2).

Eighteen (51.4%) of the studies used surveys [11,12,23-27,29-31,33-35,38,42,47,49,53], while 16 (45.7%) used either individual or focus group interviews [3,28,32,36,37,39-41,43-46,50-52,54]. One (2.9%) study used both a survey and an interview [48]. Participants were all individuals who worked clinically within a mental health care setting and included psychiatrists (n=20, 57.1%) [3,11,12,24-27,31,33,34,37-39,42-44,50,51], psychologists (n=14, 40.0%) [3,11,12,23,27,28,31,34,37,39,42,44,45,50], counselors or therapists (n=14, 40.0%) [3,12,31,34,35,37,39,41,42,45-47,49,53], nurses (n=9, 25.7%) [11,12,28,32,40,43,44,52,54], social workers or welfare officers (n=8, 22.9%) [3,12,28,37,42,46,50,54], medical doctors and general practitioners (n=7, 20.0%) [11,12,23,26,36,48,51], support workers (n=4, 11.4%) [3,12,37,42], allied health staff (n=2, 5.7%) [12,23], and other mental health professionals (unspecified; n=2, 5.7%) [11,42] (Multimedia Appendix 2).

The majority of studies discussed AI broadly (n=14, 40.0%) [11,12,23,25,26,29,32,33,35,42,51-54] or in terms of generative AI (GenAI) or large language models (LLMs) such as ChatGPT [55], Gemini [56], or Copilot [57] (n=14, 40.0%) [3,12,27,30,37-44,46,49]. Studies also discussed clinical decision support tools (eg, AI-supported diagnostic, treatment planning, risk detection, or triaging tools; n=12, 34.3%) [24,26,28,32,34,36,40,44,48,50-52], psychotherapy chatbots or conversational agents (n=9, 25.7%) [3,12,24,30,31,37,39,45,49], documentation or notetaking tools (n=4, 11.4%) [31,37,41,46], tools providing supervision or feedback on clinicians’ performance (n=4, 11.4%) [24,31,46,47], or symptom monitoring or prediction tools (n=3, 8.6%) [32,34,44] (Multimedia Appendix 2).

### Factors Shaping Clinicians’ Acceptability of AI

#### Overview

Across the included studies, clinicians’ acceptability of AI was generally conditional and context-dependent, based on how AI was intended to be used, the degree of clinician oversight retained, and the perceived level of clinical risk [3,24,36,41,44]. Clinicians tended to be more receptive to AI when it was positioned as a supportive or supplementary tool, particularly where professional judgment and responsibility remained with the clinician. Greater caution was evident where AI was perceived to operate more autonomously, replace aspects of human care, or influence higher-risk clinical decisions [41,45,49,52]. Acceptability was also influenced by clinicians’ familiarity with AI, perceived usefulness and safety, confidence in evaluating outputs, and clarity regarding professional roles and accountability [24,31,53,54].

**Figure 3.**
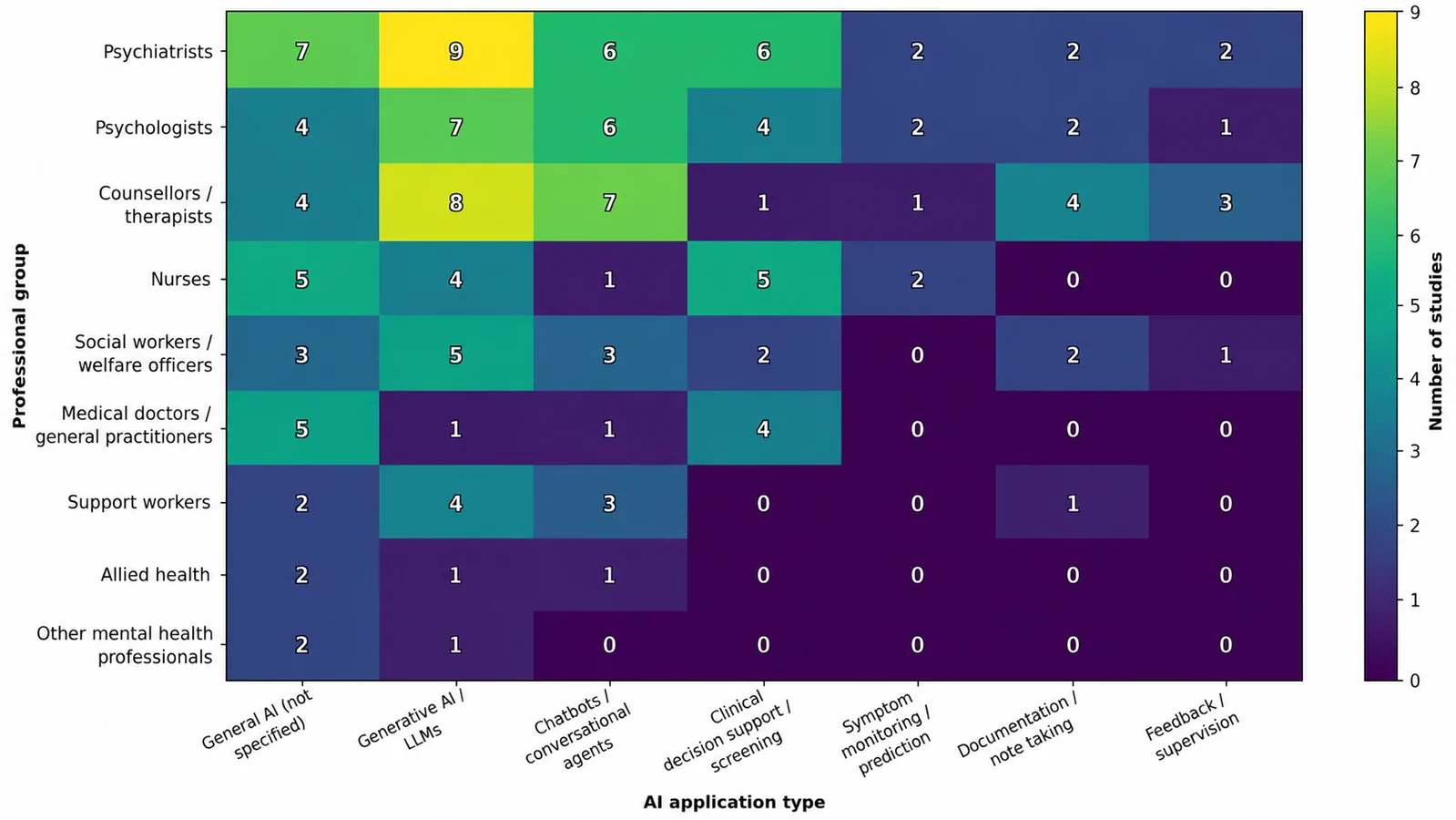
Heatmap of the distribution of charted evidence across professional groups and AI application types. LLM: large language model.

The findings charted from each included study are presented in Multimedia Appendix 3. For each study, the table summarizes clinicians’ AI knowledge and previous use, overall attitudes and perceptions, perceived benefits and risks, acceptable or proposed applications, implementation considerations, effects on professional roles and therapeutic relationships, and identified education, policy, and governance requirements. Individual studies commonly contributed evidence to several of these areas. The distribution of evidence varied substantially across professional groups, AI application types, and synthesis domains. Figure 3 maps the number of included studies examining each combination of professional group and AI application type. Evidence was concentrated among psychiatrists, psychologists, and counselors or therapists, particularly in relation to general AI, generative AI or LLMs, and conversational agents. In contrast, relatively little evidence was identified for allied health professionals, support workers, or other mental health professional groups, particularly in relation to symptom monitoring or prediction, documentation tools, feedback or supervision tools, and clinical decision support applications.

**Figure 4.**
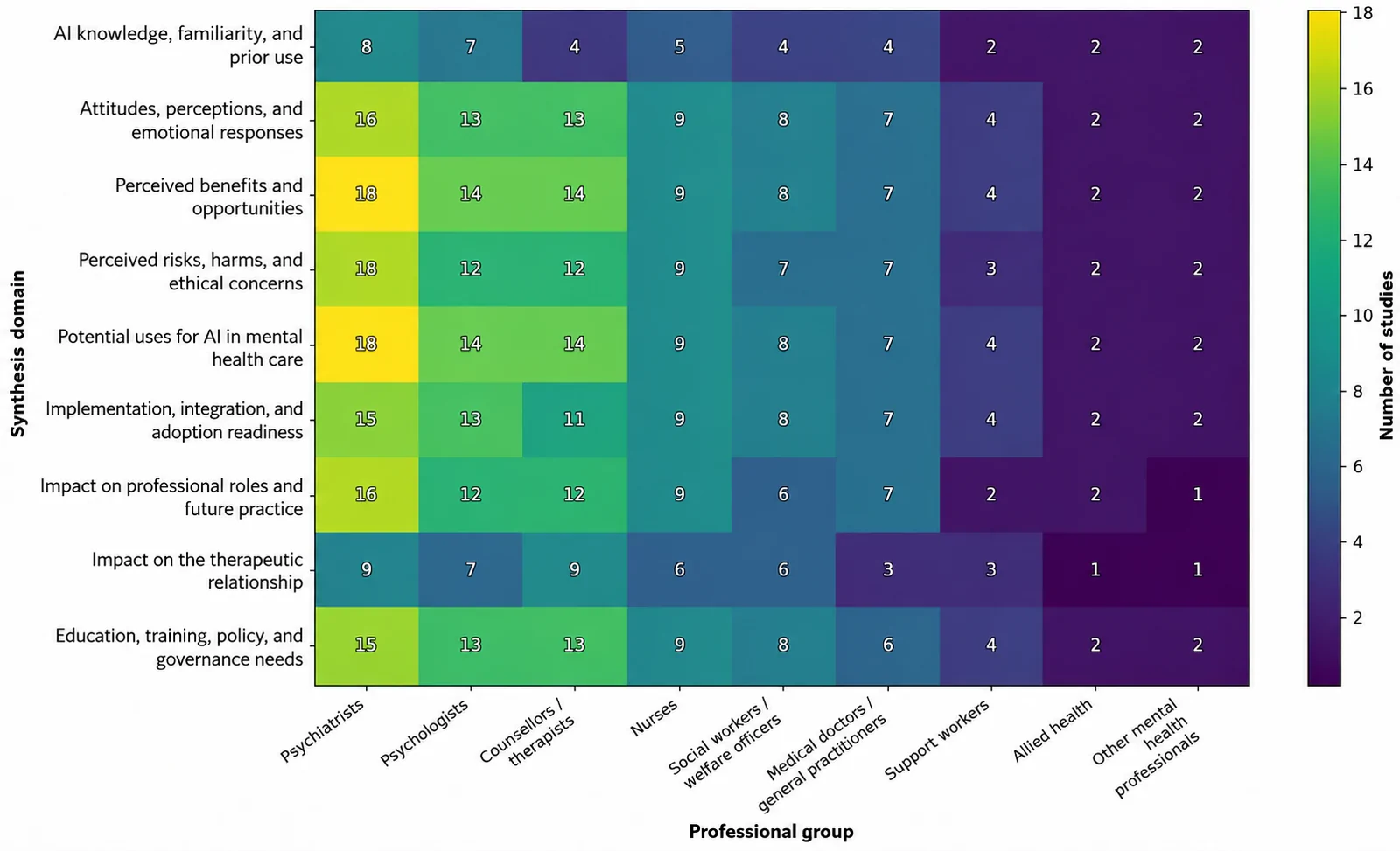
Heatmap of the distribution of charted evidence across professional groups and synthesis domains.

Figure 4 presents the distribution of charted findings across professional groups and the nine synthesis domains. Evidence was most extensive for psychiatrists, psychologists, and counselors or therapists, while substantially fewer studies contributed findings for allied health professionals, support workers, and other mental health professionals. Across professional groups, evidence relating to AI knowledge and prior use and the therapeutic relationship was generally less extensive than evidence concerning perceived benefits, potential uses, attitudes, risks, implementation, and governance.

#### AI Knowledge, Familiarity, and Prior Use

Studies demonstrated a consistent gap between awareness of AI and prior experiences with clinical AI-enabled tools. Clinicians frequently reported awareness of AI as an emerging technology; however, many had limited training or direct usage and were uncertain about how AI systems functioned or how they could safely be embedded into clinical workflows [23,24,31,54]. Clinicians demonstrated greater familiarity with GenAI tools such as ChatGPT [55]; however, previous usage was mostly limited to administrative tasks, writing summary documents, brainstorming, or general information-seeking rather than assisting with diagnosis, treatment planning, or risk assessment [12,30,37,38,41].

Studies also highlighted the overlap between clinicians’ and patients’ knowledge, familiarity, and use of AI. Even when clinicians were not personally using AI tools, they often encountered patients who were using chatbots or GenAI tools to interpret symptoms, seek reassurance, or prepare for appointments [12,30,42,45]. Clinicians raised concerns that publicly available AI tools used by patients could provide inaccurate, out-of-context, or unsafe advice, particularly when used by people who are distressed or experiencing complex mental health conditions [3,45,49]. Therefore, clinicians highlighted a need to know enough about AI to be able to ask patients about their AI usage, correct misinformation, and discuss limitations of these tools [12,30,42,45].

#### Attitudes, Perceptions, and Emotional Responses

Attitudes toward AI were generally conditional, task-dependent, and shaped by clinicians’ perceptions of control, safety, and clinical relevance [3,11,12,24,30,31,36,37,39-42,48-50,53,54]. In general, AI was perceived more positively when it was framed as complementing, not replacing clinicians [11,12,24,26,36,39,54]. Counselors and psychotherapists reported curious optimism about the potential of AI to act as an adjunct to therapy; however, they were concerned about diminishing human connection, lack of privacy, and potential bias [35,41,45,49,53]. Mental health nurses expressed greater concern for AI’s ability to understand human experience, given the human nature of mental health nursing where clinical judgment depends on emotional intelligence, contextual understanding, and ongoing patient interactions. Despite participants’ lack of knowledge and limited use of AI tools, clinicians were cautiously optimistic about its potential to enhance and streamline patient assessment processes and support risk detection; however, more education, training, and guidelines around its usage were desired before adoption into mental health care could be considered [23,24,31,32,54].

#### Perceived Benefits and Opportunities

AI was perceived as beneficial when framed as a clinical support tool that would assist in making mental health care more efficient, informed, and accessible [3,11,12,24,30,31,36,37,39,42,43,47,52-54]. Clinicians identified the potential alleviation of administrative burdens as one of the greatest benefits to the usage of AI, as it was reported to be useful in drafting or summarizing case notes, preparing documentation, and planning client sessions [12,30,37,42,46]. Clinicians also perceived AI to be beneficial in managing large volumes of patient data due to its ability to quickly synthesize information from multiple data sources, and saw potential in its ability to support screening, triage, and ongoing patient monitoring by identifying patterns that may otherwise be missed [24,34,36,54]. AI was also seen to have the potential to improve access and patients’ engagement with care. Several studies suggested that AI could support workforce shortages and unmet demand for services by supplementing care between sessions or when waitlists were long [12,24,32,39,43,44].

#### Perceived Risks, Harms, and Ethical Concerns

Despite the identified benefits of AI, clinicians were concerned about its usage and raised a number of potential risks. Most prominent was the current lack of ethics, governance, and guidelines around data privacy [3,12,32,37,39,41,43,44,51,52,54]. Given the sensitivity of mental health data, participants questioned how this information would be stored and secured, who would hold ownership of the data, and whether AI would introduce new complexities around confidentiality and enhanced data privacy [12,39,54].

Clinicians also raised concerns about clinical safety, quality, and limitations of AI-based systems, and whether or not these systems were at risk of providing inaccurate and insensitive responses, leading to greater risk of safety and ethical concerns, and reduced treatment effectiveness [3,24,32,43,45,47,52]. Participants also highlighted that AI may not have the ability to consider the nuances of complex human behavior observed in psychiatry, and would struggle to interpret severe emotional states, interpret subtle interpersonal cues, and appropriately respond to complex, high-risk scenarios [3,43,52]. These concerns were particularly pronounced when clinicians discussed AI as providing therapeutic advice, interpreting emotional distress, supporting risk assessment, or responding directly to vulnerable clients, especially in contexts where human judgment, relational understanding, and clinical accountability were considered central to safe care [3,32,37,41-43,45,49,51,52]. Concerns about bias were also reported in several studies, including algorithmic bias, discrimination, black-box decision making, and the risk that unrepresentative training data could produce poorer outputs for minority populations [35,39,47,50]. However, concerns about bias were not consistent across studies, with some clinicians viewing the potential for AI to instead reduce bias in clinical decision making [24,36,44].

Participants raised concerns about overreliance on AI and unclear role boundaries, worrying that clinicians or clients may place too much trust in AI outputs, especially where systems appeared knowledgeable, authoritative, or empathic [30,41,42,45,47]. Clinicians also questioned whether responsibility for AI-associated harm would rest with patients, clinicians, health care organizations, or AI developers [12,37,41,44,51]. This lack of clarity was central to clinicians’ reluctance to support integration of AI, and reinforced the need for transparency, clear governance, and human oversight.

#### Potential Uses for AI in Mental Health Care

The included studies identified a range of potential uses for AI in mental health care, though perceived acceptability varied. Administrative and clinician-facing applications were consistently deemed the most acceptable, and were viewed to be relatively low risk as they reduced administrative burden while preserving clinicians’ focus for patient-facing, clinical tasks. These applications include documentation, case note generation, report writing, session summaries, and reflective practice [12,30,37,42,46].

Screening, triage, monitoring, and decision-making support tools were also discussed as promising applications [24,32,34,36,43,48,54]. For instance, clinicians felt AI would be useful to synthesize large volumes of information, identify clinical patterns, support screening or risk detection, and provide prompts or recommendations within a clinician-facing application [24,32,34,36,43,44,48,50,54]. By contrast, clinicians were more cautious about AI applications involving diagnostic or treatment decisions, crisis response, therapeutic interactions, or those that are patient-facing [3,26,41,45,49,52]. Some studies suggested that AI tools may have potential as adjunctive supports for psychoeducation, between-session support, homework tasks, or improving access to information, provided that they are used carefully and with appropriate safeguards [3,37,45].

#### Implementation, Integration, and Adoption Readiness

Implementation and adoption readiness were shaped by both individual and organizational factors. At the individual level, clinicians’ willingness to use AI depended on perceived usefulness, AI literacy, confidence, and trust [24,31,32,53,54]. Several studies identified limited familiarity and lack of AI-related training as barriers to adoption, suggesting that clinicians may be reluctant not because they reject the use of AI, but because they feel unprepared to use, evaluate, or explain it [24,31,53,54].

At an organizational level, clinicians emphasized the need for streamlined workflow integration, organizational readiness for change, clear governance, and evidence of safety and effectiveness [31,32,36,44,54]. Studies that examined implementation considerations found that clinicians required AI tools to be integrated into existing clinical routines, rather than being an additional burden or disruption, as well as clear guidance on when AI should be used, how outputs should be interpreted, and who would remain accountable for AI-influenced decisions [36,44,54].

In some studies, clinicians voiced a desire to be involved in the integration of AI to positively guide uptake, suggesting adoption readiness may be improved through co-design, training, and transparent communication [36,54]. Overall, successful implementation appeared to be dependent on embedding AI within safe, clinician-led, organizationally-supported models of care.

#### Impact of AI on Professional Roles and Future Practice

Clinicians generally did not view AI as a replacement for mental health professionals, but many expected it to reshape several aspects of clinical practice [25,26,29,30,33,35,39,46,47]. AI was deemed most likely to impact administrative, documentation, and information processing tasks [25,26,29,30,33], while assessment, therapeutic engagement, and professional judgment were likely to remain unchanged [25,26,29,33,35]. Nevertheless, some clinicians remained concerned that AI could devalue professional expertise, reduce recognition of human skills, or be promoted as a cheaper substitute for clinical care [25,32,41,47,52]. However, others felt that AI could strengthen professional practice by reducing administrative burden, supporting reflection, improving efficiency, and contributing to training or supervision [35,46,47].

#### Impact on the Therapeutic Relationship

The therapeutic relationship was repeatedly identified as a central issue in clinicians’ perceptions of AI. Mental health nursing staff, in particular, questioned whether or not it was appropriate to use AI to address patients’ clinical needs, and felt that AI could not adequately meet patients’ needs for emotional support, empathy, and face-to-face communication [52]. Clinicians emphasized that mental health care relies on human contact, compassion, and therapeutic alliance, and felt that patients may prefer to be treated by a human rather than by AI [29,32,35,41,45,49,52].

At the same time, AI was not always viewed as harmful to the therapeutic relationship. Some clinicians highlighted that AI could indirectly support patient-clinician relationships by reducing administrative burdens and allowing clinicians to be more present with clients, or by providing a level of support to clients in between sessions [45,46]. Nursing staff also identified potential roles for AI to assist in care planning, patient monitoring, risk detection, and improving access to information, while maintaining that AI should support, rather than replace, human-centered care [52].

#### Education, Training, Policy, and Governance Needs

Clinicians called for education and training on how AI systems work, their functionalities, how to interpret outputs, and how risks such as bias, hallucinations, privacy breaches, and overreliance should be managed. Training needs were closely linked to adoption readiness, with clinicians indicating greater willingness to engage with AI when they understood its limitations, had opportunities to practice using it, and could clearly see how it fit within their professional role [24,31,53,54]. Clinicians who highlighted that patients were already using AI tools recognized an additional need to be able to discuss AI use with patients to guide safe and appropriate engagement. Clinicians called for clear governance and policies that provide ethical guidelines, privacy safeguards, professional standards, regulatory oversight, and organizational policies clarifying accountability [11,23,24,32,37,43,44,49,54]. Safe structures, education, and governance were not seen as optional additions to the implementation of AI in mental health care. Rather, they were perceived as central requirements necessary to build clinicians’ trust and confidence, protect patients, and ensure that AI is used in a safe and supportive manner [11,23,24,31,32,37,38,43,44,53,54].

## Discussion

### General Discussion

This scoping review aimed to synthesize the current evidence on clinicians’ attitudes, perceptions, and beliefs around the use of AI in mental health care. The findings indicate that clinicians are cautiously optimistic about AI, but their acceptance is conditional on how AI is used. Clinicians recognized potential value in AI tools, which could reduce administrative burden, support documentation, assist with screening or monitoring, and improve access to care, but raised concerns about privacy, clinical safety, accountability, professional role boundaries, and impacts on the therapeutic relationship. Overall, clinicians do not necessarily reject AI in mental health care, but successful implementation requires clear role boundaries, human oversight, education, governance, and evidence of safe use. These findings are consistent with broader health care AI literature, which argues that the translation of AI into clinical practice depends on more than model performance and requires workflow integration, clinician trust, clear accountability, and prospective evaluations in real-world settings [58,59].

The mapping of the evidence demonstrated that the literature is currently unevenly distributed across professional groups, AI applications, and areas of clinician experience. Evidence was concentrated among psychiatrists, psychologists, and counselors or therapists, whereas comparatively few studies included allied health professionals, support workers, or other mental health professional groups. This imbalance is important because different professional groups have distinct roles, scopes of practice, patterns of patient contact, and responsibilities within mental health services, and may therefore experience the introduction of AI in different ways. Similarly, some AI applications, including symptom monitoring and prediction, documentation, and feedback or supervision tools, were examined across a narrower range of professional groups than general AI, GenAI, and conversational agents. The domain-level mapping further demonstrated comparatively limited evidence regarding clinicians’ AI knowledge and prior use and the impact of AI on therapeutic relationships. These gaps suggest that the existing literature may provide a stronger understanding of clinicians’ general attitudes toward AI, rather than of how specific technologies are experienced by the diverse workforce expected to use them in practice. Future research should therefore broaden representation across professional groups and examine specific AI applications within the clinical contexts in which they are intended to be implemented, with particular attention to clinicians’ real-world experience with these technologies and their effects on therapeutic care.

Successful implementation of AI into mental health care should begin with clinical need, rather than technological capability. Clinicians were most receptive to AI when it addressed current pressures on mental health services, such as administrative burden, workforce constraints, the abundance of information, and delays in patients’ access to care, suggesting that implementation should prioritize tools that address clearly defined problems within existing models of care. Introducing AI simply because the technology is available risks the introduction of tools that are poorly aligned to clinical workflows, increase burden, or fail to address issues within mental health practice, reflecting wider implementation concerns [58]. Lower-risk clinician-facing applications, such as documentation support, may provide a practical starting point to build clinicians’ knowledge and familiarity with AI tools, while preserving more time and attention for human interaction and clinical judgment [58-60].

To best support clinicians’ usage of AI, education should be treated as a prerequisite for successful implementation, rather than optional additions [61,62]. Clinicians require training that extends beyond surface-level familiarity to include the ability to generate prompts, critically appraise outputs, communicate with patients about appropriate AI use, understand privacy and confidentiality, recognize bias or hallucinations, manage overreliance, and be aware of accountability [63-65]. Moving forward, training programs, such as microcredentials, will be imperative for clinicians to develop core competencies required to safely use AI in mental health care. Legal responsibility and accountability also require careful consideration. Broader health care–AI literature has highlighted that existing liability frameworks may be poorly suited to address harms associated with AI, particularly where responsibility may be distributed across clinicians, health care organizations, software developers, service providers, and regulators [66,67]. Implementation will require clear governance, defining the intended roles of AI tools, clinicians’ responsibilities in checking outputs, documentation requirements, escalation pathways, and processes for monitoring errors or adverse events [59,61].

This review also highlights the need for co-design and real-world evaluation, as clinicians voiced a desire to be involved in AI implementation. Co-design is critical in mental health care as the consequences of poor implementation extend beyond technical errors and may affect patient safety, equity, therapeutic relationships, and trust [59,61,62,68]. This aligns with co-design literature in mental health care, which argues that interventions should be designed and evaluated with, rather than for, the people who will use them [68]. A tool that appears accurate in controlled testing environments may still not be successful if it is disruptive to therapeutic relationships, increases administrative burden, produces inaccurate outputs, or is perceived to be replacing human elements of care. AI development and implementation should be guided not only by clinicians but also by other key stakeholders including patients, carers, developers, researchers, and policymakers, to ensure that they are usable, acceptable, and clinically appropriate. Current AI evaluation guidance emphasizes the need to assess human-AI interactions, workflow integration, safety, and usability rather than technical performance alone [59,61,62,69].

### Limitations

As a scoping review, this study aims to map the existing literature rather than critically appraise study quality or establish causal relationships, which limits the ability to draw conclusions about the effectiveness or impact of AI-based tools in clinical practice. The review is also constrained by the scope and quality of the included studies, many of which rely on self-reported attitudes and perceptions that are susceptible to response bias, social desirability effects, and variations in AI literacy among clinicians. Additionally, the rapidly evolving nature of generative AI means that clinicians’ views captured in the literature may quickly become outdated as tools, governance frameworks, and clinical integration mature. Finally, heterogeneity in study designs, clinical settings, professional roles, and definitions of AI may limit the generalizability of the findings across different mental health contexts and health systems.

### Conclusions

This review is innovative in shifting the focus from the technical performance of AI tools to the perspectives of the clinicians who will be expected to use, interpret, explain, and remain accountable for them in mental health care. Unlike previous reviews, this review provides a clinician-centered understanding of AI adoption, highlighting that acceptability depends not only on what AI can do but also on whether it can be integrated safely, ethically, and in ways that preserve professional judgment and therapeutic relationships. These findings suggest that implementation should begin with lower-risk (eg, administrative), clinician-facing applications, be supported by education and governance, and be evaluated in mental health settings before wider adoption.

Since the release of ChatGPT [55] in 2022, public awareness of AI-enabled tools has increased rapidly, prompting broader reflection on where these systems can and should be used. AI is rapidly becoming ubiquitous in health care, and as a result, mental health services will increasingly need to determine not only whether AI tools can be used but whether they can be implemented safely, ethically, and in ways that preserve clinicians’ judgment and patients’ trust. For this reason, future research should move to examine and evaluate how AI tools function in real-world mental health settings. This should include assessment of clinical workflow, clinician workload, patient experience, equity, privacy, safety, therapeutic relationships, and unintended consequences over time. Integration of AI into mental health care will therefore require ongoing evaluation and refinement, rather than one-off validation before implementation. The future of implementation into mental health care will therefore depend on ensuring that AI strengthens services without eroding human judgment, trust, and therapeutic relationships that remain central.

## Supplementary material

10.2196/92370 Multimedia Appendix 1 Search strategy.

10.2196/92370 Multimedia Appendix 2 Study characteristics.

10.2196/92370 Multimedia Appendix 3 Key findings from included studies.

10.2196/92370 Checklist 1 PRISMA-ScR checklist.

10.2196/92370 Checklist 2 PRISMA-S checklist.

## Abbreviations

GenAI: generative AI
JBI: Joanna Briggs Institute
LLM: large language model
PRISMA-S: Preferred Reporting Items for Systematic Reviews and Meta-Analyses Literature Search Extension
PRISMA-ScR: Preferred Reporting Items for Systematic Reviews and Meta-Analyses Extension for Scoping Reviews

## References

[1] Xie Y, Zhai Y, Lu G (2024). Evolution of artificial intelligence in healthcare: a 30-year bibliometric study. Front Med (Lausanne).

[2] Hudson C, Goldsworthy A, Phan TL, Joy A, Tronstad O, Randall M (2026). Use of natural language processing in the emergency department: a clinical overview on the state of the art. Artif Intell Health.

[3] Hipgrave L, Goldie J, Dennis S, Coleman A (2025). Balancing risks and benefits: clinicians’ perspectives on the use of generative AI chatbots in mental healthcare. Front Digit Health.

[4] Burns R, Graff K (2021). Theories of Psychotherapy and Counseling: Concepts and Cases.

[5] Geldard D, Geldard K, Foo RY (2017). Basic Personal Counselling: A Training Manual for Counsellors.

[6] Putica A, Khanna R, Bosl W, Saraf S, Edgcomb J (2025). Ethical decision-making for AI in mental health: the Integrated Ethical Approach for Computational Psychiatry (IEACP) framework. Psychol Med.

[7] Tavory T (2024). Regulating AI in mental health: ethics of care perspective. JMIR Ment Health.

[8] Peek N, Capurro D, Rozova V, van der Veer SN (2024). Bridging the gap: challenges and strategies for the implementation of artificial intelligence-based clinical decision support systems in clinical practice. Yearb Med Inform.

[9] Saeidnia HR, Hashemi Fotami SG, Lund B, Ghiasi N (2024). Ethical considerations in artificial intelligence interventions for mental health and well-being: ensuring responsible implementation and impact. Soc Sci.

[10] Scipion CEA, Manchester MA, Federman A, Wang Y, Arias JJ (2025). Barriers to and facilitators of clinician acceptance and use of artificial intelligence in healthcare settings: a scoping review. BMJ Open.

[11] Sharif L, Almabadi R, Alahmari A (2025). Perceptions of mental health professionals towards artificial intelligence in mental healthcare: a cross-sectional study. Front Psychiatry.

[12] Cross S, Bell I, Nicholas J (2024). Use of AI in mental health care: community and mental health professionals survey. JMIR Ment Health.

[13] Cruz-Gonzalez P, He AWJ, Lam EP (2025). Artificial intelligence in mental health care: a systematic review of diagnosis, monitoring, and intervention applications. Psychol Med.

[14] He Y, Yang L, Qian C (2023). Conversational agent interventions for mental health problems: systematic review and meta-analysis of randomized controlled trials. J Med Internet Res.

[15] Li H, Zhang R, Lee YC, Kraut RE, Mohr DC (2023). Systematic review and meta-analysis of AI-based conversational agents for promoting mental health and well-being. NPJ Digit Med.

[16] Young AT, Amara D, Bhattacharya A, Wei ML (2021). Patient and general public attitudes towards clinical artificial intelligence: a mixed methods systematic review. Lancet Digit Health.

[17] Henzler D, Schmidt S, Koçar A (2025). Healthcare professionals’ perspectives on artificial intelligence in patient care: a systematic review of hindering and facilitating factors on different levels. BMC Health Serv Res.

[18] Rebelo AD, Verboom DE, dos Santos NR, de Graaf JW (2023). The impact of artificial intelligence on the tasks of mental healthcare workers: a scoping review. Comput Hum Behav Artif Humans.

[19] Tricco AC, Lillie E, Zarin W (2018). PRISMA Extension for Scoping Reviews (PRISMA-ScR): checklist and explanation. Ann Intern Med.

[20] Peters MDJ, Marnie C, Tricco AC (2020). Updated methodological guidance for the conduct of scoping reviews. JBI Evid Synth.

[21] Hudson C, Phan TL, Randall M (2026). Research protocol: adoption of AI in mental health clinical decision support: a scoping review of clinician attitudes and perceptions. Open Source Framework (OSF).

[22] Covidence.

[23] Aamer I, Tariq K, Rashid A, Haider II (2025). Mental health professionals’ perspectives on artificial intelligence in mental health services: a cross-sectional study in Pakistan. Ann King Edw Med Univ.

[24] Abiodun OA, Ajiboye PO, Salihu MO (2025). Psychiatrists’ and trainees’ knowledge, perception, and readiness for integration of artificial intelligence in mental health care in Nigeria. BMC Psychiatry.

[25] Al-Ansari AM, Al-Medfa MK (2023). Psychiatrists’ attitudes toward artificial intelligence: tasks, job security and benefits. Bahrain Med Bull.

[26] Alsalman Z (2026). Artificial intelligence in mental health care: task-specific perspectives of professionals in Saudi Arabia. Healthcare (Basel).

[27] Aral A, Gerdan G, Usta MB, Aral AE (2025). From promise to practice: insights into ChatGPT-4o use in child and adolescent mental health from professionals. Front Psychiatry.

[28] Auf H, Nygren J, Lundgren LE, Petersson L, Svedberg P (2025). Healthcare professionals’ perspectives on AI-driven decision support in young adult mental health: an analysis through the lens of a shared decision-making framework. Front Digit Health.

[29] Blease C, Locher C, Leon-Carlyle M, Doraiswamy M (2020). Artificial intelligence and the future of psychiatry: qualitative findings from a global physician survey. Digit Health.

[30] Blease C, Worthen A, Torous J (2024). Psychiatrists’ experiences and opinions of generative artificial intelligence in mental healthcare: an online mixed methods survey. Psychiatry Res.

[31] Cecil J, Kleine AK, Lermer E, Gaube S (2025). Mental health practitioners’ perceptions and adoption intentions of AI-enabled technologies: an international mixed-methods study. BMC Health Serv Res.

[32] Dong J, Chen X, Lyu C (2026). Attitudes of psychiatric nurses towards the integration of artificial intelligence applications to clinical care: a qualitative study in China. BMC Nurs.

[33] Doraiswamy PM, Blease C, Bodner K (2020). Artificial intelligence and the future of psychiatry: insights from a global physician survey. Artif Intell Med.

[34] Fischer L, Mann PA, Nguyen MHH (2025). AI for mental health: clinician expectations and priorities in computational psychiatry. BMC Psychiatry.

[35] Fulmer R, Zhai Y, Beeson ET (2026). Counsellors attitudes, emotions, and ethical concerns regarding artificial intelligence: results from a professional survey. Couns Psychother Res.

[36] Ghadiri P, Yaffe MJ, Adams AM, Abbasgholizadeh-Rahimi S (2024). Primary care physicians’ perceptions of artificial intelligence systems in the care of adolescents’ mental health. BMC Prim Care.

[37] Goldie J, Dennis S, Hipgrave L, Coleman A (2025). Practitioner perspectives on the uses of generative AI chatbots in mental health care: mixed methods study. JMIR Hum Factors.

[38] Gruber EN, Zlatec LG, Biočina SM (2025). Awareness and practice of using public generative AI solutions (such as ChatGPT) and social media among psychiatrists compared to other professionals: a pilot study. Psychiatr Danub.

[39] Gültekin M, Şahin M (2024). The use of artificial intelligence in mental health services in Turkey: what do mental health professionals think?. Cyberpsychology.

[40] Günday EA, Güler KG (2026). Artificial intelligence through the eyes of psychiatric nurses: an in-depth investigation of thought, anxiety and readiness. Issues Ment Health Nurs.

[41] Kuang J, Pope AL, Zhang Y (2026). Psychotherapists' trust, distrust, and generative AI practices in psychotherapy: qualitative study. J Med Internet Res.

[42] Linardon J, Liu C, Messer M, McClure Z, Anderson C, Jarman HK (2025). Current practices and perspectives of artificial intelligence in the clinical management of eating disorders: insights from clinicians and community participants. Int J Eat Disord.

[43] Ma Y, Zeng Y, Liu T, Sun R, Xiao M, Wang J (2024). Integrating large language models in mental health practice: a qualitative descriptive study based on expert interviews. Front Public Health.

[44] Maas J, Franssen S, Petkovic M (2026). Artificial intelligence in eating disorder treatment: a qualitative analysis of clinical opportunities, barriers, and ethical considerations from multi-disciplinary focus groups. Int J Eat Disord.

[45] Mackey A (2026). Psychotherapists’ perspectives on AI chatbots as adjuncts to psychotherapy: opportunities and concerns. Couns Psychother Res.

[46] Matthews EB, Lerman D, Beach N, Wiczyk D, Goldkind L (2026). “It’s like having that supervisor in the room”: examining AI as a reflective partner. Psychother Res.

[47] Moran LH, Kee SC, Wiese CW, Arriaga RI, Abdullah S, Sherrill AM (2025). Artificial intelligence as a feedback teammate for treatment delivery: cognitive behavioral therapists’ hopes and fears. Cogn Behav Pract.

[48] Popescu C, Golden G, Benrimoh D (2021). Evaluating the clinical feasibility of an artificial intelligence-powered, web-based clinical decision support system for the treatment of depression in adults: longitudinal feasibility study. JMIR Form Res.

[49] Prescott J, Hanley T (2023). Therapists’ attitudes towards the use of AI in therapeutic practice: considering the therapeutic alliance. Ment Health Soc Incl.

[50] A SS, Rao A (2026). Exploring mental health professionals’ perceptions and acceptance of AI-based screening tools. IIUM Med J Malays.

[51] Stroud AM, Curtis SH, Weir IB (2025). Physician perspectives on the potential benefits and risks of applying artificial intelligence in psychiatric medicine: qualitative study. JMIR Ment Health.

[52] Tuncer GZ, Çetinkaya Duman Z (2025). The exploration of psychiatric nurses' perspectives on the applications of artificial intelligence in supporting care: 'patients prefer seeing a human over AI'. J Psychiatr Ment Health Nurs.

[53] Wagner J, Schwind AS (2025). Investigating psychotherapists’ attitudes towards artificial intelligence in psychotherapy. BMC Psychol.

[54] Zhang M, Scandiffio J, Younus S (2023). The adoption of AI in mental health care-perspectives from mental health professionals: qualitative descriptive study. JMIR Form Res.

[55] ChatGPT.

[56] Google Gemini.

[57] Microsoft Copilot.

[58] Kelly CJ, Karthikesalingam A, Suleyman M, Corrado G, King D (2019). Key challenges for delivering clinical impact with artificial intelligence. BMC Med.

[59] Vasey B, Nagendran M, Campbell B (2022). Reporting guideline for the early stage clinical evaluation of decision support systems driven by artificial intelligence: DECIDE-AI. BMJ.

[60] Topol EJ (2019). High-performance medicine: the convergence of human and artificial intelligence. Nat Med.

[61] (2021). Ethics and governance of artificial intelligence for health. https://iris.who.int/server/api/core/bitstreams/f780d926-4ae3-42ce-a6d6-e898a5562621/content.

[62] (2023). Regulatory considerations on artificial intelligence for health. https://iris.who.int/server/api/core/bitstreams/ad62580f-540f-4e36-b957-e7f2946ae1fb/content.

[63] Amann J, Blasimme A, Vayena E, Frey D, Madai VI, Precise4Q consortium (2020). Explainability for artificial intelligence in healthcare: a multidisciplinary perspective. BMC Med Inform Decis Mak.

[64] Meskó B (2023). Prompt engineering as an important emerging skill for medical professionals: tutorial. J Med Internet Res.

[65] Meskó B, Görög M (2020). A short guide for medical professionals in the era of artificial intelligence. NPJ Digit Med.

[66] Gerke S, Minssen T, Cohen G, Bohr A, Memarzadeh K (2020). Artificial Intelligence in Healthcare.

[67] Maliha G, Gerke S, Cohen IG, Parikh RB (2021). Artificial intelligence and liability in medicine: balancing safety and innovation. Milbank Q.

[68] Stiles-Shields C, Cummings C, Montague E, Plevinsky JM, Psihogios AM, Williams KDA (2022). A call to action: using and extending human-centered design methodologies to improve mental and behavioral health equity. Front Digit Health.

[69] Liu X, Cruz Rivera S, Moher D, Calvert MJ, Denniston AK, SPIRIT-AI and CONSORT-AI Working Group (2020). Reporting guidelines for clinical trial reports for interventions involving artificial intelligence: the CONSORT-AI extension. Lancet Digit Health.

