# Supplementary material for: Clinicians’ Attitudes and Perceptions on the Adoption of AI in Mental Health Care: Scoping Review
Source: J Med Internet Res. 2026 Sep 11;28:e92370. doi: 10.2196/92370 (PMC13566077; doi:10.2196/92370)
Supplement: Supplementary file 2 [file jmir-v28-e92370-s005.pdf]

PRISMA-S Checklist

Section/topic	#	Checklist item	Location(s) Reported
**INFORMATION SOURCES AND METHODS**			
Database name	1	Name each individual database searched, stating the platform for each.	Information Sources
Multi-database searching	2	If databases were searched simultaneously on a single platform, state the name of the platform, listing all of the databases searched.	Information Sources
Study registries	3	List any study registries searched.	Information Sources
Online resources and browsing	4	Describe any online or print source purposefully searched or browsed (e.g., tables of contents, print conference proceedings, web sites), and how this was done.	Information Sources
Citation searching	5	Indicate whether cited references or citing references were examined, and describe any methods used for locating cited/citing references (e.g., browsing reference lists, using a citation index, setting up email alerts for references citing included studies).	Information Sources
Contacts	6	Indicate whether additional studies or data were sought by contacting authors, experts, manufacturers, or others.	Information Sources
Other methods	7	Describe any additional information sources or search methods used.	Information Sources
**SEARCH STRATEGIES**			
Full search strategies	8	Include the search strategies for each database and information source, copied and pasted exactly as run.	Appendix A
Limits and restrictions	9	Specify that no limits were used, or describe any limits or restrictions applied to a search (e.g., date or time period, language, study design) and provide justification for their use.	Information Sources
Search filters	10	Indicate whether published search filters were used (as originally designed or modified), and if so, cite the filter(s) used.	Information Sources

Prior work	11	Indicate when search strategies from other literature reviews were adapted or reused for a substantive part or all of the search, citing the previous review(s).	Search strategy
Updates	12	Report the methods used to update the search(es) (e.g., rerunning searches, email alerts).	Information sources
Dates of searches	13	For each search strategy, provide the date when the last search occurred.	Appendix A
**PEER REVIEW**			
Peer review	14	Describe any search peer review process.	Search strategy
**MANAGING RECORDS**			
Total Records	15	Document the total number of records identified from each database and other information sources.	Figure 1
Deduplication	16	Describe the processes and any software used to deduplicate records from multiple database searches and other information sources.	Screening and Selection Process

PRISMA-S: An Extension to the PRISMA Statement for Reporting Literature Searches in Systematic Reviews
Rethlefsen ML, Kirtley S, Waffenschmidt S, Ayala AP, Moher D, Page MJ, Koffel JB, PRISMA-S Group.
Last updated February 27, 2020.
