# Supplementary material for: Clinicians’ Attitudes and Perceptions on the Adoption of AI in Mental Health Care: Scoping Review
Source: J Med Internet Res. 2026 Sep 11;28:e92370. doi: 10.2196/92370 (PMC13566077; doi:10.2196/92370)
Supplement: Supplementary file 3 [file jmir-v28-e92370-s001.docx]

# **Appendix A – Search Strategies**

***Limited to 2020 onwards***

**PubMED**

("Health Personnel"[Mesh] OR "Physicians"[Mesh] OR "Nurses"[Mesh] OR "Psychiatrists"[Mesh] OR "Psychologists"[Mesh] OR "Social Workers"[Mesh] OR "Occupational Therapists"[Mesh] OR "General Practitioners"[Mesh] OR "Physicians, Family"[Mesh] OR "Family Practice"[Mesh] OR clinician*[tiab] OR "health personnel"[tiab] OR "health professional*"[tiab] OR "healthcare professional*"[tiab] OR "health care professional*"[tiab] OR "healthcare worker*"[tiab] OR "health care worker*"[tiab] OR physician*[tiab] OR doctor*[tiab] OR nurs*[tiab] OR psychiatrist*[tiab] OR psychologist*[tiab] OR psychotherapist*[tiab] OR therapist*[tiab] OR counsellor*[tiab] OR counselor*[tiab] OR "social worker*"[tiab] OR "case manager*"[tiab] OR "peer worker*"[tiab] OR "peer support worker*"[tiab] OR "mental health professional*"[tiab] OR "mental health practitioner*"[tiab] OR "mental health clinician*"[tiab] OR "psychiatric nurse*"[tiab] OR "clinical psychologist*"[tiab] OR "general practitioner*"[tiab] OR GP[tiab] OR GPs[tiab] OR "allied health"[tiab])

AND

("Artificial Intelligence"[Mesh] OR "Machine Learning"[Mesh] OR "Deep Learning"[Mesh] OR "Natural Language Processing"[Mesh] OR "Decision Support Systems, Clinical"[Mesh] OR "artificial intelligence"[tiab] OR AI[tiab] OR "machine learning"[tiab] OR NLP[tiab] OR "clinical decision support"[tiab] OR "decision support system*"[tiab] OR CDSS[tiab] OR "AI-enabled"[tiab] OR "AI-based"[tiab] OR "AI-assisted"[tiab] OR "AI-supported"[tiab] OR "algorithmic decision support"[tiab] OR chatbot*[tiab] OR "conversational agent*"[tiab] OR "virtual agent*"[tiab] OR "digital assistant*"[tiab] OR "generative AI"[tiab] OR "generative artificial intelligence"[tiab] OR "large language model*"[tiab] OR LLM[tiab] OR LLMs[tiab] OR ChatGPT[tiab] OR "GPT-3"[tiab] OR "GPT-4"[tiab] OR "GPT-4o"[tiab] OR “GPT-5”[tiab] OR Gemini[tiab] OR Bard[tiab] OR Copilot[tiab] OR "Bing Chat"[tiab] OR "Med-PaLM"[tiab] OR "Med-PaLM 2"[tiab])

AND

("Attitude of Health Personnel"[Mesh] OR "Health Knowledge, Attitudes, Practice"[Mesh] OR "Diffusion of Innovation"[Mesh] OR "User-Computer Interface"[Mesh] OR "Qualitative Research"[Mesh] OR "Surveys and Questionnaires"[Mesh] OR "Interviews as Topic"[Mesh] OR "Focus Groups"[Mesh] OR attitude*[tiab] OR perception*[tiab] OR perspective*[tiab] OR opinion*[tiab] OR belief*[tiab] OR view*[tiab] OR experience*[tiab] OR knowledge[tiab] OR awareness[tiab] OR acceptab*[tiab] OR acceptance[tiab] OR "technology acceptance"[tiab] OR adoption[tiab] OR uptake[tiab] OR readiness[tiab] OR preparedness[tiab] OR "intention to use"[tiab] OR willingness[tiab] OR trust[tiab] OR confidence[tiab] OR usability[tiab] OR feasibility[tiab] OR appropriateness[tiab] OR satisfaction[tiab] OR concern*[tiab] OR barrier*[tiab] OR facilitator*[tiab] OR challenge*[tiab] OR risk*[tiab] OR benefit*[tiab] OR implement*[tiab])

AND

("Mental Health Services"[Mesh] OR "Community Mental Health Services"[Mesh] OR "Emergency Services, Psychiatric"[Mesh] OR "Psychiatry"[Mesh] OR "Mental Disorders"[Mesh] OR "Psychotherapy"[Mesh] OR psychiat*[tiab] OR psychiatric[tiab] OR "mental health"[tiab] OR "mental healthcare"[tiab] OR "mental health care"[tiab] OR "behavioral health"[tiab] OR "behavioural health"[tiab] OR psychotherap*[tiab] OR psycholog*[tiab] OR "psychological therap*"[tiab] OR "psychiatric ward*"[tiab] OR "psychiatric unit*"[tiab] OR "psychiatric hospital*"[tiab] OR "community mental health"[tiab] OR "acute psychiatric"[tiab] OR "inpatient psychiatric"[tiab] OR "outpatient psychiatric"[tiab])

**CINAHL**

(MH "Health Personnel+" OR MH "Physicians+" OR MH "Nurses+" OR MH "Social Workers" OR MH "Occupational Therapists" OR TI clinician* OR AB clinician* OR TI "health personnel" OR AB "health personnel" OR TI "health professional*" OR AB "health professional*" OR TI "healthcare professional*" OR AB "healthcare professional*" OR TI "health care professional*" OR AB "health care professional*" OR TI "healthcare worker*" OR AB "healthcare worker*" OR TI "health care worker*" OR AB "health care worker*" OR TI physician* OR AB physician* OR TI doctor* OR AB doctor* OR TI nurs* OR AB nurs* OR TI psychiatrist* OR AB psychiatrist* OR TI psychologist* OR AB psychologist* OR TI psychotherapist* OR AB psychotherapist* OR TI therapist* OR AB therapist* OR TI counsellor* OR AB counsellor* OR TI counselor* OR AB counselor* OR TI "social worker*" OR AB "social worker*" OR TI "case manager*" OR AB "case manager*" OR TI "peer worker*" OR AB "peer worker*" OR TI "peer support worker*" OR AB "peer support worker*" OR TI "mental health professional*" OR AB "mental health professional*" OR TI "mental health practitioner*" OR AB "mental health practitioner*" OR TI "mental health clinician*" OR AB "mental health clinician*" OR TI "psychiatric nurse*" OR AB "psychiatric nurse*" OR TI "clinical psychologist*" OR AB "clinical psychologist*" OR TI "general practitioner*" OR AB "general practitioner*" OR TI GP OR AB GP OR TI GPs OR AB GPs OR TI "allied health" OR AB "allied health")

AND

(MH "Artificial Intelligence+" OR MH "Decision Support Systems, Clinical" OR TI "artificial intelligence" OR AB "artificial intelligence" OR TI AI OR AB AI OR TI "machine learning" OR AB "machine learning" OR TI "deep learning" OR AB "deep learning" OR TI "natural language processing" OR AB "natural language processing" OR TI NLP OR AB NLP OR TI "clinical decision support" OR AB "clinical decision support" OR TI "decision support system*" OR AB "decision support system*" OR TI CDSS OR AB CDSS OR TI "AI-enabled" OR AB "AI-enabled" OR TI "AI-based" OR AB "AI-based" OR TI "AI-assisted" OR AB "AI-assisted" OR TI "AI-supported" OR AB "AI-supported" OR TI "algorithmic decision support" OR AB "algorithmic decision support" OR TI chatbot* OR AB chatbot* OR TI "conversational agent*" OR AB "conversational agent*" OR TI "virtual agent*" OR AB "virtual agent*" OR TI "digital assistant*" OR AB "digital assistant*" OR TI "generative AI" OR AB "generative AI" OR TI "generative artificial intelligence" OR AB "generative artificial intelligence" OR TI "large language model*" OR AB "large language model*" OR TI LLM OR AB LLM OR TI LLMs OR AB LLMs OR TI ChatGPT OR AB ChatGPT OR TI "GPT-3" OR AB "GPT-3" OR TI "GPT-4" OR AB "GPT-4" OR TI "GPT-4o" OR AB "GPT-4o" OR TI "GPT-5" OR AB "GPT-5" OR TI Gemini OR AB Gemini OR TI Bard OR AB Bard OR TI Copilot OR AB Copilot OR TI "Bing Chat" OR AB "Bing Chat" OR TI "Med-PaLM" OR AB "Med-PaLM" OR TI "Med-PaLM 2" OR AB "Med-PaLM 2")

AND

(MH "Attitude of Health Personnel+" OR MH "Health Knowledge, Attitudes, Practice" OR MH "Surveys" OR MH "Interviews" OR MH "Focus Groups" OR TI attitude* OR AB attitude* OR TI perception* OR AB perception* OR TI perspective* OR AB perspective* OR TI opinion* OR AB opinion* OR TI belief* OR AB belief* OR TI view* OR AB view* OR TI experience* OR AB experience* OR TI knowledge OR AB knowledge OR TI awareness OR AB awareness OR TI acceptab* OR AB acceptab* OR TI acceptance OR AB acceptance OR TI "technology acceptance" OR AB "technology acceptance" OR TI adoption OR AB adoption OR TI uptake OR AB uptake OR TI readiness OR AB readiness OR TI preparedness OR AB preparedness OR TI "intention to use" OR AB "intention to use" OR TI willingness OR AB willingness OR TI trust OR AB trust OR TI confidence OR AB confidence OR TI usability OR AB usability OR TI feasibility OR AB feasibility OR TI appropriateness OR AB appropriateness OR TI satisfaction OR AB satisfaction OR TI concern* OR AB concern* OR TI barrier* OR AB barrier* OR TI facilitator* OR AB facilitator* OR TI challenge* OR AB challenge* OR TI risk* OR AB risk* OR TI benefit* OR AB benefit* OR TI implement* OR AB implement*)

AND

(MH "Mental Health Services+" OR MH "Community Mental Health Services" OR MH "Psychiatry" OR MH "Mental Disorders+" OR MH "Psychotherapy+" OR TI psychiat* OR AB psychiat* OR TI psychiatric OR AB psychiatric OR TI "mental health" OR AB "mental health" OR TI "mental healthcare" OR AB "mental healthcare" OR TI "mental health care" OR AB "mental health care" OR TI "behavioral health" OR AB "behavioral health" OR TI "behavioural health" OR AB "behavioural health" OR TI psychotherap* OR AB psychotherap* OR TI psycholog* OR AB psycholog* OR TI "psychological therap*" OR AB "psychological therap*" OR TI "psychiatric ward*" OR AB "psychiatric ward*" OR TI "psychiatric unit*" OR AB "psychiatric unit*" OR TI "psychiatric hospital*" OR AB "psychiatric hospital*" OR TI "community mental health" OR AB "community mental health" OR TI "acute psychiatric" OR AB "acute psychiatric" OR TI "inpatient psychiatric" OR AB "inpatient psychiatric" OR TI "outpatient psychiatric" OR AB "outpatient psychiatric")

**EMBASE**

('health care personnel' OR 'physician'/exp OR 'nurse'/exp OR 'psychiatrist'/exp OR 'psychologist'/exp OR 'social worker'/exp OR 'occupational therapist'/exp OR 'general practitioner'/exp OR clinician*:ti,ab,kw OR 'health professional*':ti,ab,kw OR 'healthcare professional*':ti,ab,kw OR 'health care professional*':ti,ab,kw OR psychiatrist*:ti,ab,kw OR psychologist*:ti,ab,kw OR psychotherapist*:ti,ab,kw OR counsellor*:ti,ab,kw OR counselor*:ti,ab,kw OR 'social worker*':ti,ab,kw OR 'case manager*':ti,ab,kw OR 'peer worker*':ti,ab,kw OR 'peer support worker*':ti,ab,kw OR 'mental health professional*':ti,ab,kw OR 'mental health practitioner*':ti,ab,kw OR 'mental health clinician*':ti,ab,kw OR 'psychiatric nurse*':ti,ab,kw OR 'clinical psychologist*':ti,ab,kw OR 'general practitioner*':ti,ab,kw OR 'allied health':ti,ab,kw)

AND

('artificial intelligence'/exp OR 'machine learning'/exp OR 'deep learning'/exp OR 'natural language processing'/exp OR 'clinical decision support system'/exp OR 'chatbot'/exp OR 'artificial intelligence':ti,ab,kw OR ai:ti,ab,kw OR 'machine learning':ti,ab,kw OR 'deep learning':ti,ab,kw OR 'natural language processing':ti,ab,kw OR nlp:ti,ab,kw OR 'clinical decision support':ti,ab,kw OR 'decision support system*':ti,ab,kw OR cdss:ti,ab,kw OR 'ai-enabled':ti,ab,kw OR 'ai-based':ti,ab,kw OR 'ai-assisted':ti,ab,kw OR 'ai-supported':ti,ab,kw OR 'algorithmic decision support':ti,ab,kw OR chatbot*:ti,ab,kw OR 'conversational agent*':ti,ab,kw OR 'virtual agent*':ti,ab,kw OR 'digital assistant*':ti,ab,kw OR 'generative ai':ti,ab,kw OR 'generative artificial intelligence':ti,ab,kw OR 'large language model*':ti,ab,kw OR llm:ti,ab,kw OR llms:ti,ab,kw OR chatgpt:ti,ab,kw OR 'gpt-3':ti,ab,kw OR 'gpt-4':ti,ab,kw OR 'gpt-4o':ti,ab,kw OR 'gpt-5':ti,ab,kw OR gemini:ti,ab,kw OR bard:ti,ab,kw OR copilot:ti,ab,kw OR 'bing chat':ti,ab,kw OR 'med-palm':ti,ab,kw OR 'med-palm 2':ti,ab,kw)

AND

('attitude to health'/exp OR 'health knowledge attitude practice'/exp OR 'technology adoption'/exp OR 'qualitative research'/exp OR 'questionnaire'/exp OR 'interview'/exp OR 'focus group'/exp OR attitude*:ti,ab,kw OR perception*:ti,ab,kw OR perspective*:ti,ab,kw OR opinion*:ti,ab,kw OR belief*:ti,ab,kw OR view*:ti,ab,kw OR experience*:ti,ab,kw OR knowledge:ti,ab,kw OR awareness:ti,ab,kw OR acceptab*:ti,ab,kw OR acceptance:ti,ab,kw OR 'technology acceptance':ti,ab,kw OR adoption:ti,ab,kw OR uptake:ti,ab,kw OR readiness:ti,ab,kw OR preparedness:ti,ab,kw OR 'intention to use':ti,ab,kw OR willingness:ti,ab,kw OR trust:ti,ab,kw OR confidence:ti,ab,kw OR usability:ti,ab,kw OR feasibility:ti,ab,kw OR appropriateness:ti,ab,kw OR satisfaction:ti,ab,kw OR concern*:ti,ab,kw OR barrier*:ti,ab,kw OR facilitator*:ti,ab,kw OR challenge*:ti,ab,kw OR risk*:ti,ab,kw OR benefit*:ti,ab,kw OR implement*:ti,ab,kw)

AND

('mental health service'/exp OR 'community mental health care'/exp OR 'psychiatry'/exp OR 'psychotherapy' OR psychiat*:ti,ab,kw OR psychiatric:ti,ab,kw OR 'mental health':ti,ab,kw OR 'mental healthcare':ti,ab,kw OR 'mental health care':ti,ab,kw OR 'behavioral health':ti,ab,kw OR 'behavioural health':ti,ab,kw OR psychotherap*:ti,ab,kw OR 'psychiatric ward*':ti,ab,kw OR 'psychiatric unit*':ti,ab,kw OR 'psychiatric hospital*':ti,ab,kw OR 'community mental health':ti,ab,kw OR 'acute psychiatric':ti,ab,kw OR 'inpatient psychiatric':ti,ab,kw OR 'outpatient psychiatric':ti,ab,kw)

**PsycINFO**

1. exp Mental Health Personnel/ OR exp Psychiatrists/ OR exp Psychologists/ OR exp Psychotherapists/ OR exp Psychiatric Nurses/ OR exp Social Workers/ OR exp Occupational Therapists/ OR exp General Practitioners/ OR clinician*.ti,ab,id. OR health professional*.ti,ab,id. OR healthcare professional*.ti,ab,id. OR health care professional*.ti,ab,id. OR psychiatrist*.ti,ab,id. OR psychologist*.ti,ab,id. OR psychotherapist*.ti,ab,id. OR counsellor*.ti,ab,id. OR counselor*.ti,ab,id. OR social worker*.ti,ab,id. OR case manager*.ti,ab,id. OR peer worker*.ti,ab,id. OR peer support worker*.ti,ab,id. OR mental health professional*.ti,ab,id. OR mental health practitioner*.ti,ab,id. OR mental health clinician*.ti,ab,id. OR psychiatric nurse*.ti,ab,id. OR clinical psychologist*.ti,ab,id. OR general practitioner*.ti,ab,id. OR allied health.ti,ab,id.

2. exp Artificial Intelligence/ OR exp Machine Learning/ OR exp Deep Learning/ OR exp Natural Language Processing/ OR exp Decision Support Systems/ OR exp Chatbots/ OR artificial intelligence.ti,ab,id. OR AI.ti,ab,id. OR machine learning.ti,ab,id. OR deep learning.ti,ab,id. OR natural language processing.ti,ab,id. OR NLP.ti,ab,id. OR clinical decision support.ti,ab,id. OR decision support system*.ti,ab,id. OR CDSS.ti,ab,id. OR AI-enabled.ti,ab,id. OR AI-based.ti,ab,id. OR AI-assisted.ti,ab,id. OR AI-supported.ti,ab,id. OR algorithmic decision support.ti,ab,id. OR chatbot*.ti,ab,id. OR conversational agent*.ti,ab,id. OR virtual agent*.ti,ab,id. OR digital assistant*.ti,ab,id. OR generative AI.ti,ab,id. OR generative artificial intelligence.ti,ab,id. OR large language model*.ti,ab,id. OR LLM.ti,ab,id. OR LLMs.ti,ab,id. OR ChatGPT.ti,ab,id. OR GPT-3.ti,ab,id. OR GPT-4.ti,ab,id. OR GPT-4o.ti,ab,id. OR GPT-5.ti,ab,id. OR Gemini.ti,ab,id. OR Bard.ti,ab,id. OR Copilot.ti,ab,id. OR Bing Chat.ti,ab,id. OR Med-PaLM.ti,ab,id. OR Med-PaLM 2.ti,ab,id.

3. exp Attitudes/ OR exp Professional Attitudes/ OR exp Knowledge Level/ OR exp Health Knowledge/ OR exp Technology Adoption/ OR exp Qualitative Research/ OR exp Questionnaires/ OR exp Interviews/ OR exp Focus Groups/ OR attitude*.ti,ab,id. OR perception*.ti,ab,id. OR perspective*.ti,ab,id. OR opinion*.ti,ab,id. OR belief*.ti,ab,id. OR view*.ti,ab,id. OR experience*.ti,ab,id. OR knowledge.ti,ab,id. OR awareness.ti,ab,id. OR acceptab*.ti,ab,id. OR acceptance.ti,ab,id. OR "technology acceptance".ti,ab,id. OR adoption.ti,ab,id. OR uptake.ti,ab,id. OR readiness.ti,ab,id. OR preparedness.ti,ab,id. OR "intention to use".ti,ab,id. OR willingness.ti,ab,id. OR trust.ti,ab,id. OR confidence.ti,ab,id. OR usability.ti,ab,id. OR feasibility.ti,ab,id. OR appropriateness.ti,ab,id. OR satisfaction.ti,ab,id. OR concern*.ti,ab,id. OR barrier*.ti,ab,id. OR facilitator*.ti,ab,id. OR challenge*.ti,ab,id. OR risk*.ti,ab,id. OR benefit*.ti,ab,id. OR implement*.ti,ab,id.

4. exp Mental Health Services/ OR exp Community Mental Health Services/ OR exp Psychiatry/ OR exp Mental Disorders/ OR exp Psychotherapy/ OR psychiat*.ti,ab,id. OR psychiatric.ti,ab,id. OR mental health.ti,ab,id. OR mental healthcare.ti,ab,id. OR mental health care.ti,ab,id. OR behavioral health.ti,ab,id. OR behavioural health.ti,ab,id. OR psychotherap*.ti,ab,id. OR psychiatric ward*.ti,ab,id. OR psychiatric unit*.ti,ab,id. OR psychiatric hospital*.ti,ab,id. OR community mental health.ti,ab,id. OR acute psychiatric.ti,ab,id. OR inpatient psychiatric.ti,ab,id. OR outpatient psychiatric.ti,ab,id.

5. 1 AND 2 AND 3 AND 4

**Scopus**

TITLE-ABS-KEY(clinician* OR "health professional*" OR "healthcare professional*" OR "health care professional*" OR psychiatrist* OR psychologist* OR psychotherapist* OR counsellor* OR counselor* OR "social worker*" OR "case manager*" OR "peer worker*" OR "peer support worker*" OR "mental health professional*" OR "mental health practitioner*" OR "mental health clinician*" OR "psychiatric nurse*" OR "clinical psychologist*" OR "general practitioner*" OR "allied health")

AND

TITLE-ABS-KEY("artificial intelligence" OR ai OR "machine learning" OR "deep learning" OR "natural language processing" OR nlp OR "clinical decision support" OR "decision support system*" OR "AI-enabled" OR "AI-based" OR "AI-assisted" OR "AI-supported" OR "algorithmic decision support" OR chatbot* OR "conversational agent*" OR "virtual agent*" OR "digital assistant*" OR "generative AI" OR "generative artificial intelligence" OR "large language model*" OR llm OR llms OR ChatGPT OR "GPT-3" OR "GPT-4" OR "GPT-4o" OR "GPT-5" OR Gemini OR Bard OR Copilot OR "Bing Chat" OR "Med-PaLM" OR "Med-PaLM 2")

AND

TITLE-ABS-KEY(attitude* OR perception* OR perspective* OR opinion* OR belief* OR view* OR experience* OR knowledge OR awareness OR acceptab* OR acceptance OR "technology acceptance" OR adoption OR uptake OR readiness OR preparedness OR "intention to use" OR willingness OR trust OR confidence OR usability OR feasibility OR appropriateness OR satisfaction OR concern* OR barrier* OR facilitator* OR challenge* OR risk* OR benefit* OR implement*)

AND

TITLE-ABS-KEY(psychiat* OR psychiatric OR "mental health" OR "mental healthcare" OR "mental health care" OR "behavioral health" OR "behavioural health" OR psychotherap* OR "psychiatric ward*" OR "psychiatric unit*" OR "psychiatric hospital*" OR "community mental health" OR "acute psychiatric" OR "inpatient psychiatric" OR "outpatient psychiatric")

**Web of Science**

TS=(clinician* OR "health professional*" OR "healthcare professional*" OR "health care professional*" OR psychiatrist* OR psychologist* OR psychotherapist* OR counsellor* OR counselor* OR "social worker*" OR "case manager*" OR "peer worker*" OR "peer support worker*" OR "mental health professional*" OR "mental health practitioner*" OR "mental health clinician*" OR "psychiatric nurse*" OR "clinical psychologist*" OR "general practitioner*" OR "allied health")

AND

TS=("artificial intelligence" OR AI OR "machine learning" OR "deep learning" OR "natural language processing" OR NLP OR "clinical decision support" OR "decision support system*" OR CDSS OR "AI-enabled" OR "AI-based" OR "AI-assisted" OR "AI-supported" OR "algorithmic decision support" OR chatbot* OR "conversational agent*" OR "virtual agent*" OR "digital assistant*" OR "generative AI" OR "generative artificial intelligence" OR "large language model*" OR LLM OR LLMs OR ChatGPT OR "GPT-3" OR "GPT-4" OR "GPT-4o" OR "GPT-5" OR Gemini OR Bard OR Copilot OR "Bing Chat" OR "Med-PaLM" OR "Med-PaLM 2")

AND

TS=(attitude* OR perception* OR perspective* OR opinion* OR belief* OR view* OR experience* OR knowledge OR awareness OR acceptab* OR acceptance OR "technology acceptance" OR adoption OR uptake OR readiness OR preparedness OR "intention to use" OR willingness OR trust OR confidence OR usability OR feasibility OR appropriateness OR satisfaction OR concern* OR barrier* OR facilitator* OR challenge* OR risk* OR benefit* OR implement*)

AND

TS=(psychiat* OR psychiatric OR "mental health" OR "mental healthcare" OR "mental health care" OR "behavioral health" OR "behavioural health" OR psychotherap* OR "psychiatric ward*" OR "psychiatric unit*" OR "psychiatric hospital*" OR "community mental health" OR "acute psychiatric" OR "inpatient psychiatric" OR "outpatient psychiatric")
