# Supplementary material for: Clinicians’ Attitudes and Perceptions on the Adoption of AI in Mental Health Care: Scoping Review
Source: J Med Internet Res. 2026 Sep 11;28:e92370. doi: 10.2196/92370 (PMC13566077; doi:10.2196/92370)
Supplement: Supplementary file 4 [file jmir-v28-e92370-s002.docx]

**Appendix 2.** Study Characteristics

| **Citation** | **Year** | **Country** | **Method** | | **Participants’ Occupation** | | | | | | | | | **Type of AI** | | | | | | |  |
| --- | --- | --- | --- | --- | --- | --- | --- | --- | --- | --- | --- | --- | --- | --- | --- | --- | --- | --- | --- | --- | --- |
|  |  |  | **Survey** | **Focus groups /interviews** | **Psychiatrists** | **Psychologists** | **Counsellors/ therapists** | **Nurses** | **Social workers/welfare officers** | **Medical doctors/ general practitioners** | **Support workers** | **Allied health** | **Other mental health professionals (not specified)** | **Generative AI/LLMs** | **AI chatbots/ conversational agents** | **Clinical decision support/screening** | **Symptom monitoring/ prediction** | **Documentation/ notetaking** | **Feedback/supervision tools** | **General AI (not specified)** | |
| Aamer et al. | 2025 | Pakistan | ✔ |  |  | ✔ |  |  |  | ✔ |  | ✔ |  |  |  |  |  |  |  | ✔ | |
| Abiodun et al. | 2025 | Nigeria | ✔ |  | ✔ |  |  |  |  |  |  |  |  |  |  | ✔ | ✔ |  |  | ✔ | |
| Al-Ansari & Al-Medfa | 2023 | Bahrain | ✔ |  | ✔ |  |  |  |  |  |  |  |  |  |  |  |  |  |  | ✔ | |
| Alsalman | 2026 | Saudi Arabia | ✔ |  | ✔ |  |  |  |  | ✔ |  |  |  |  |  | ✔ |  |  |  | ✔ | |
| Aral et al. | 2025 | Turkey | ✔ |  | ✔ | ✔ |  |  |  |  |  |  |  | ✔ |  |  |  |  |  |  | |
| Auf et al. | 2025 | Sweden |  | ✔ |  | ✔ |  | ✔ | ✔ |  |  |  |  |  |  | ✔ |  |  |  |  | |
| Blease et al. | 2020 | Global | ✔ |  | ✔ |  |  |  |  |  |  |  |  |  |  |  |  |  |  | ✔ | |
| Blease et al. | 2024 | USA | ✔ |  | ✔ |  |  |  |  |  |  |  |  | ✔ | ✔ |  |  |  |  |  | |
| Cecil et al. | 2025 | Germany and USA | ✔ |  | ✔ | ✔ | ✔ |  |  |  |  |  |  |  |  | ✔ |  |  | ✔ | ✔ | |
| Cross et al. | 2024 | Australia | ✔ |  | ✔ | ✔ | ✔ | ✔ | ✔ | ✔ | ✔ | ✔ |  | ✔ | ✔ |  |  |  |  | ✔ | |
| Dong et al. | 2026 | China |  | ✔ |  |  |  | ✔ |  |  |  |  |  |  |  | ✔ | ✔ |  |  | ✔ | |
| Doraiswamy et al. | 2020 | Global | ✔ |  | ✔ |  |  |  |  |  |  |  |  |  |  |  |  |  |  | ✔ | |
| Fischer et al. | 2025 | Germany | ✔ |  | ✔ | ✔ | ✔ |  |  |  |  |  |  |  |  | ✔ | ✔ |  |  |  | |
| Fulmer et al. | 2026 | USA | ✔ |  |  |  | ✔ |  |  |  |  |  |  |  |  |  |  |  |  | ✔ | |
| Ghadiri et al. | 2024 | Canada |  | ✔ |  |  |  |  |  | ✔ |  |  |  |  |  | ✔ |  |  |  |  | |
| Goldie et al. | 2025 | Australia |  | ✔ | ✔ | ✔ | ✔ |  | ✔ |  | ✔ |  |  | ✔ | ✔ |  |  | ✔ |  |  | |
| Gruber et al. | 2025 | Global | ✔ |  | ✔ |  |  |  |  |  |  |  |  | ✔ |  |  |  |  |  |  | |
| Gultekin & Sahin | 2024 | Turkey |  | ✔ | ✔ | ✔ | ✔ |  |  |  |  |  |  | ✔ | ✔ |  |  |  |  |  | |
| Gunday et al. | 2025 | Turkey |  | ✔ |  |  |  | ✔ |  |  |  |  |  | ✔ |  | ✔ |  |  |  |  | |
| Hipgrave et al. | 2025 | Australia |  | ✔ | ✔ | ✔ | ✔ |  | ✔ |  | ✔ |  |  | ✔ | ✔ |  |  |  |  |  | |
| Kuang et al. | 2026 | USA |  | ✔ |  |  | ✔ |  |  |  |  |  |  | ✔ |  |  |  | ✔ |  |  | |
| Linardon et al. | 2025 | Global | ✔ |  | ✔ | ✔ | ✔ |  | ✔ |  | ✔ |  | ✔ | ✔ |  |  |  |  |  | ✔ | |
| Ma et al. | 2024 | China |  | ✔ | ✔ |  |  | ✔ |  |  |  |  |  | ✔ |  |  |  |  |  |  | |
| Maas et al. | 2026 | The Netherlands |  | ✔ | ✔ | ✔ |  | ✔ |  |  |  |  |  | ✔ |  | ✔ | ✔ |  |  |  | |
| Mackey et al. | 2026 | Republic of Ireland |  | ✔ |  | ✔ | ✔ |  |  |  |  |  |  |  | ✔ |  |  |  |  |  | |
| Matthews et al. | 2025 | USA |  | ✔ |  |  | ✔ |  | ✔ |  |  |  |  | ✔ |  |  |  | ✔ | ✔ |  | |
| Moran et al. | 2025 | USA | ✔ |  |  |  | ✔ |  |  |  |  |  |  |  |  |  |  |  | ✔ |  | |
| Popescu et al. | 2021 | Canada | ✔ | ✔ |  |  |  |  |  | ✔ |  |  |  |  |  | ✔ |  |  |  |  | |
| Prescott and Hanley | 2023 | UK | ✔ |  |  |  | ✔ |  |  |  |  |  |  | ✔ | ✔ |  |  |  |  |  | |
| Sharif et al. | 2025 | Saudi Arabia | ✔ |  | ✔ | ✔ |  | ✔ |  | ✔ |  |  | ✔ |  |  |  |  |  |  | ✔ | |
| Shetty & Rao | 2026 | India |  | ✔ | ✔ | ✔ |  |  | ✔ |  |  |  |  |  |  | ✔ |  |  |  |  | |
| Stroud et al. | 2025 | USA |  | ✔ | ✔ |  |  |  |  | ✔ |  |  |  |  |  | ✔ |  |  |  | ✔ | |
| Tuncer & Cetinkaya Duman | 2025 | Turkey |  | ✔ |  |  |  | ✔ |  |  |  |  |  |  |  | ✔ |  |  |  | ✔ | |
| Wagner & Schwind | 2025 | Germany | ✔ |  |  |  | ✔ |  |  |  |  |  |  |  |  |  |  |  |  | ✔ | |
| Zhang et al. | 2023 | Canada |  | ✔ |  |  |  | ✔ | ✔ |  |  |  |  |  |  |  |  |  |  | ✔ | |
