# Supplementary material for: Clinicians’ Attitudes and Perceptions on the Adoption of AI in Mental Health Care: Scoping Review
Source: J Med Internet Res. 2026 Sep 11;28:e92370. doi: 10.2196/92370 (PMC13566077; doi:10.2196/92370)
Supplement: Supplementary file 5 [file jmir-v28-e92370-s003.docx]

**Appendix 3 – Key Findings from Included Studies**

| **Citation and Year** | **AI knowledge, familiarity, and prior use** | **Attitudes, perceptions, and emotional responses** | **Perceived benefits and opportunities** | **Perceived risks, harms, and ethical concerns** | **Potential uses for AI in mental health care** | **Implementation, integration, and adoption readiness** | **Impact on professional roles and future practice** | **Impact on the therapeutic relationship** | **Education, training, policy, and governance needs** |
| --- | --- | --- | --- | --- | --- | --- | --- | --- | --- |
| **Aamer et al. (2025)** | High awareness; little formal training; chatbots were the most recognised AI tool. | Cautious optimism. | Reduced workload and improved access to mental health services. | Ethical concerns and doubts about diagnostic accuracy. | Workplace tasks and personal wellbeing support; use was preferred to supplement rather than replace standard care. | Human oversight was preferred. | AI was not viewed as a replacement for clinicians. | Not reported. | Formal training, ethical safeguards, and regulation. |
| **Abiodun et al. (2025)** | Most were aware of AI’s potential, although familiarity and prior use were limited; many reported readiness to adopt AI. | Most held positive views of AI integration. | Diagnostic assistance, monitoring, outcome prediction, personalised and standardised care, reduced bias, and improved access. | Data security, loss of human interaction, reduced empathy, and inability to perform nuanced mental status assessment. | Diagnosis support, patient monitoring, outcome prediction, and care planning. | Many expressed willingness to adopt AI; cost of implementation was a concern. | AI was considered valuable for addressing service gaps but unsuitable for replacing complex clinical judgement. | Loss of human interaction and reduced empathy were concerns; AI was considered unsuitable for replacing empathy. | Targeted training and ethical regulation. |
| **Al-Ansari & Al-Medfa (2023)** | Not reported. | Concern about job security and the possible replacement of psychiatric tasks. | Faster diagnostic process. | Concern that AI could replace physician roles or human skills. | Prognosis, synthesis of information for diagnosis, and obtaining medical or psychiatric histories. | Not reported. | Concern that AI could replace physician roles or human skills; some examination tasks were viewed as less likely to be replaced. | Empathic care was viewed as less likely to be replaced. | Not reported. |
| **Alsalman (2026)** | Not reported. | AI was viewed as a supportive technology that would change professional roles rather than replace clinicians. | Increased efficiency and automation of administrative tasks. | Concerns were greater for higher-risk decisions requiring contextual judgement, accountability, and empathy. | Clinical documentation, diagnostic or assessment support; high-risk suicide assessment was considered less appropriate. | Clinician-led integration with clear task boundaries. | AI was expected to change professional roles rather than replace clinicians; human clinicians remained central for ethical judgement and high-risk care. | Human clinicians remained central for empathy. | Ethically grounded integration. |
| **Aral et al. (2025)** | Prior use was reported by both psychiatrists and psychologists. | Both professional groups were moderately positive. | Potential for clinician-facing support, psychoeducation, improved access to care, self-help, and crisis support. | Ethical concerns, bias, and uncertainty regarding patient-facing and therapist-like roles. | Clinician-facing and patient-facing tools, psychoeducation, crisis planning, and diagnostic or treatment support. | Cautious openness to adoption; psychiatrists showed somewhat greater willingness to use ChatGPT-4o than psychologists. Both groups prioritised ethics and system oversight before clinical integration. | Professional groups differed in their comfort with specific clinical roles. | Not reported. | Ethical safeguards. |
| **Auf et al. (2025)** | Not reported. | Healthcare professionals recognised potential value but expressed conditional trust. | Earlier detection, assessment, personalised recommendations, triage, patient participation, and information exchange. | Misinterpretation of non-verbal cues, overdiagnosis, overreliance, reduced autonomy, inequity, and poor trust. | AI-supported shared decision-making, early detection, assessment, triage, and treatment recommendations. | Human-centred co-design with clinicians and patients, and integration into existing workflow processes. | AI should support rather than weaken clinician autonomy. | Concerns included weakened trust and therapeutic relationships. | Safety safeguards. |
| **Blease et al. (2020)** | Not reported. | Psychiatrists expressed divergent views and were highly skeptical that AI could replace human empathy. | Potential improvements in efficiency and access to care and reductions in cost. | Mixed views about effects on care and the profession; possible harms to patient–psychiatrist interactions and quality of care. | Clinician-AI collaboration in clinical decisions was anticipated by many participants. | Not reported. | AI was not considered able to replace psychiatrists. | Human empathy and patient-psychiatrist interactions were considered difficult or impossible for AI to replace. | Not reported. |
| **Blease et al. (2024)** | Many psychiatrists had used generative AI for clinical questions. | Opinions about its value and professional impact remained mixed. | More efficient documentation and administrative work. | Uncertainty about reliability, professional role, and patients’ independent use of generative AI. | Clinical questions, documentation, and other administrative support. | Not reported. | AI was expected to change psychiatry, but not necessarily replace psychiatrists. | Not reported. | Substantial clinician support and training were considered necessary. |
| **Cecil et al. (2025)** | Clinicians showed limited familiarity with AI. | Clinicians showed cautious, application-specific attitudes, with greater willingness to learn about AI than to use it clinically, and less hesitation toward clinician-facing than patient-facing tools. | Potential for earlier detection, more personalised treatment, practitioner feedback, and reduced administrative burden. | AI-related anxiety, insufficient familiarity, and uncertainty about ethical use. | Diagnostic, treatment, feedback, and practice-management applications. | Adoption intentions varied by learning motivation, ethical knowledge, and technology affinity. | Clinicians queried their capability of evaluating and using AI within their professional role. | Not reported. | Training should address learning intentions, ethical knowledge, technology confidence, and application-specific needs. |
| **Cross et al. (2024)** | Use of AI was increasing. | Mental health professionals were generally positive, particularly when compared to community members. | Accessibility, reduced cost, personalisation, research and writing support, and increased work efficiency. | Reduced human connection, privacy and data security, ethics, regulation, misuse, medical errors, and experienced harms. | Research, report writing, work-related support, and client-facing support. | Ongoing monitoring of benefits and harms. | AI was viewed as potentially useful but unable to replace clinicians. | Reduced human connection was a concern. | Legal and ethical safeguards, regulation, privacy protection, and data-security protections. |
| **Dong et al. (2026)** | Psychiatric nurses reported limited knowledge of AI. | Psychiatric nurses were positive but cautious. | Reduced workload, improved efficiency and safety, monitoring, early warning, documentation, risk assessment, and personalised care planning. | Privacy breaches, weak consent and accountability, loss of human-led care, misinterpretation, role ambiguity, and job-security concerns. | Assistance with ward rounds, vital-sign and sleep monitoring, records, reports, behavioural risk detection, and nursing decision support. | AI should remain supportive and operate within clearly defined tasks. | Nursing judgement was considered essential; role displacement and job security were concerns. | Loss of human-led care was a concern; empathy, companionship, and communication were considered essential. | Structured education, clinical guidelines, ethical regulation, legal responsibility, data protection, and hospital policies. |
| **Doraiswamy et al. (2020)** | Not reported. | Few psychiatrists expected AI to make their jobs obsolete, although around half expected substantial change to their work. | Documentation and synthesis of information were most often viewed as replaceable by AI. | Uncertainty about whether the benefits of AI would outweigh the risks; concern about loss of empathic care. | Documentation, updating records, and information synthesis. | Integration and need for reskilling were identified as key implementation considerations. | Few expected AI to fully replace make psychiatrists’ jobs, although around half expected substantial changes to work. | Empathic care was viewed as substantially less replaceable than administrative and information-processing tasks. | Not reported. |
| **Fischer et al. (2025)** | Not reported. | Clinicians prioritised practical, clinically actionable AI tools. | Continuous monitoring, prediction of symptom trajectories, and proactive patient monitoring. | Not reported. | Outpatient monitoring and predictive modelling using self-reports, third-party observations, and sleep data. | Development should prioritise accurate tools for real-world practice. | Clinicians prioritised predictive support over explicit treatment recommendations. | Not reported. | Not reported. |
| **Fulmer et al. (2026)** | Not reported. | Counsellors were predominantly optimistic, whilst remaining skeptical about AI’s relational and diagnostic capacity. | Pattern recognition, support tasks, reduced workload and burnout, and improved access. | Loss of human connection, privacy, confidentiality, bias, unreliable recommendations, and weak therapeutic capacity. | Supportive and administrative functions rather than independent counselling. | Not reported. | AI was seen as an adjunct, rather than a replacement, to human counsellors. | Counsellors were skeptical about AI’s relational capacity and rejected equivalence between AI interventions and human counselling. | Ethical guidance, privacy safeguards, professional training, and limits on clinical use. |
| **Ghadiri et al. (2024)** | Not reported. | Primary care clinicians were open to AI and willing to contribute to development. | Cost-effectiveness for tasks requiring administrative burden. | Loss of clinical competency and possible patient harm. | Data collection and synthesis, diagnosis support, and treatment planning for adolescent mental health. | User-friendly design, clinician co-design, and compensation for clinician input. | AI should assist rather than replace primary care judgement. | Not reported. | Regulation and clear legal and ethical guidance. |
| **Goldie et al. (2025)** | Most participants had no prior exposure to generative AI chatbots in a mental health setting; exposure to the demonstration increased their understanding of their potential applications. | Clinicians favoured administrative applications; likelihood of recommending chatbots increased after viewing a demonstration. | Reduced administrative burden. | AI was not seen as suitable for session planning. | Generating case notes. | Safe and effective integration was recommended. | Not reported. | Not reported. | Practitioner education and guidelines. |
| **Gruber et al. (2025)** | Psychiatrists showed limited knowledge and moderate trust; use did not differ substantially from other professionals. | Not reported. | Not reported. | Limited understanding and uncertain trust in publicly available generative AI. | Support for clinical decision-making, research, and education. | Not reported. | No clear replacement role was supported in the extracted findings. | Not reported. | Targeted education and continued research. |
| **Gultekin and Sahin (2024)** | Not reported. | Participants were generally positive but cautious and did not expect radical near-term transformation. | Accessibility, reduced stigma and workload, improved satisfaction, analysis, and support for diagnosis and training. | Weaker therapeutic relationships, reliability, privacy, data ownership, black-box decision-making, bias, discrimination, cost, and resistance. | Assisting with intervention, diagnosis, training, and service delivery. | Management of cost and professional resistance. | AI was considered supplementary, rather than a replacement for clinicians. | Not reported. | Ethical and legal guidance and clear data-governance. |
| **Gunday et al. (2025)** | Psychiatric nurses reported limited knowledge and readiness. | Psychiatric nurses were cautious. | Potential observation and support functions were acknowledged, but benefits were secondary to concerns. | Inability to understand human experience, superficial observation, unsafe judgements, privacy breaches, ethical dilemmas, and patient rejection. | Observation and selected support tasks. | Human supervision required with clear task definitions. | AI could not replace psychiatric nursing judgement. | AI could not replace empathy, therapeutic communication, or contextual interpretation. | Training for nurses and patients and evidence-based digital-health policies. |
| **Hipgrave et al. (2025)** | Prior experience with generative AI was limited; ChatGPT was the most commonly used tool among those with experience. | Clinicians held balanced, highly contextual views; a chatbot demonstration did not shift the perceived balance of risks and benefits. | Access, affordability, multilingual support, clients’ homework support, current information, and engagement. | Lack of regulation, privacy concerns, poor contextual understanding, overreliance, incorrect recommendations, and failure to detect subtle cues. | Setting clients’ homework, psychoeducation, multilingual and between-session support for selected groups. | Use-case and population-specific safeguards, and clinician involvement in development. | Human judgement remained necessary for contextual interpretation. | Failure to detect subtle communication cues was a concern. | Privacy protection and regulation. |
| **Kuang et al. (2026)** | Participants reported varied prior experience with GenAI, with use primarily focused on supportive, non-client-facing tasks such as documentation, brainstorming, treatment-plan wording, and resource development. | Trust was task- and context-dependent; clinicians were comfortable with low-stakes support but distrusted high-stakes uses. | Low-stakes clinician-supervised tasks and selected between-session client activities. | Loss of data control, inaccurate advice, pseudo-empathy, client attachment, avoidance of therapy, commercial incentives, and insurance pressures. | Documentation, brainstorming, treatment-goal wording, worksheets, translation, role-play, psychoeducation, training, and client preparation. | Low-stakes uses were acceptable when clinician-supervised; editable and deletable content was requested. | Clinician authority, interpretive judgement, and ethical responsibility had to remain central. | Relational primacy had to remain central; pseudo-empathy and client attachment were concerns. | Transparent data practices, organisational guidelines, and safeguards against commercial substitution of therapy. |
| **Linardon et al. (2025)** | Professional AI use was common. | Clinicians were open but cautious; enthusiasm and confidence in improved outcomes were modest. | Convenience, administrative support, continuous support, and potential workflow assistance. | Misuse, insufficient training, data privacy and security, and poor relational performance. | Administrative tasks and selected supportive applications. | Cautious implementation. | Not reported. | AI was not expected to outperform clinicians in relational skills. | Training and privacy safeguards. |
| **Ma et al. (2024)** | Not reported. | Experts recognised substantial potential but highlighted a current gap in technology within mental health care. | Improved access, patient participation, efficiency, quality, and workload allocation. | Misleading information, lack of professional nuance and depth, and risks to users. | Information support, patient engagement, and professional workflow assistance. | Patient engagement and ongoing system improvement. | AI should support rather than replace contextual clinical expertise. | Not reported. | Training and competence, usage and management guidelines, and transparency. |
| **Maas et al. (2026)** | Not reported. | Clinicians were open to AI use, especially where it reduced burden or supported monitoring. | Documentation, intake, monitoring, prediction, early identification of deterioration, between-session support, and possible reduction of human error. | Misinformation, harmful advice, over-trust, privacy, autonomy, bias, unclear responsibility, and additional workflow burden. | Session summaries, intake support, symptom monitoring, prediction, prompts during high-risk moments, and decision support. | Easy workflow access and human supervision. | AI was considered decision support only; clinicians retained judgement and responsibility. | Between-session support was considered possible, but over-trust and autonomy were concerns. | Rigorous validation, evidence of safety and reliability, training, and ethical governance. |
| **Mackey et al. (2026)** | Most participants had prior familiarity with and experience using ChatGPT. | Therapists were curious but apprehensive and supported only cautious, context-specific adjunctive use. | Could extend support between therapy sessions, provide psychoeducation and structured CBT support, and improve access for younger or digitally comfortable clients. | Dependency, isolation, reduced reflection, conflicting or incorrect advice, privacy, data ownership, weak regulation, and loss of human connection. | CBT-based work, anxiety, phobias, obsessive-compulsive symptoms, younger clients, and between-session support. | Clinical supervision and clear limits on client-facing use. | Chatbots were considered as supports, rather than replacements for therapists. | Chatbots could not replace the therapeutic alliance, authentic empathy, collaboration, or shared human experience. | Privacy and ownership safeguards and regulation. |
| **Matthews et al. (2025)** | Not reported. | Clinicians generally valued AI notetaking as both an administrative and reflective aid. | Reduced documentation burden, greater presence in sessions, reflective practice, alternative interpretations, confidence, and professional development. | Contextual errors, over-identification of risk, inaccurate diagnoses, and possible erosion of documentation and formulation skills. | Clinical note-taking, session review, reflection, case conceptualisation, and supervision-like feedback. | Outputs should be critically reviewed. | AI could support reflection but could not replace practice wisdom or professional responsibility; erosion of documentation and formulation skills was a concern. | Reduced documentation burden could create more presence during sessions; relational knowledge could not be replaced. | Training should preserve documentation and reasoning skills. |
| **Moran et al. (2025)** | Not reported. | CBT practitioners were enthusiastic about the possibility of AI providing feedback, but enthusiasm was conditional. | Treatment fidelity, therapist skill development, supervision support, flexible feedback, and potentially improved outcomes. | Confidentiality, privacy, poor feedback, disclosure to clients, ethical risks, and unproven effectiveness. | Feedback on therapy delivery, protocol adherence, and supervision of trainees. | Clinicians should be able to correct outputs; disclosure practices should be clear. | AI was envisioned as a teammate supporting rather than replacing therapist expertise. | Not reported. | Evidence of effectiveness, confidentiality safeguards, transparent feedback, and clear disclosure practices. |
| **Popescu et al. (2021)** | Patients and physicians were generally positive about the feasibility of the system. | Views on effects on the patient–clinician relationship varied. | The system was considered usable and trustworthy and did not increase appointment length. | Uncertainty about whether the system improved the patient–clinician relationship. | AI-based clinical decision support for treatment of depression. | Ease of use and fit with existing clinical workflows were assessed; further evaluation was indicated. | The system supported clinician treatment selection and management. | Participants were uncertain whether the system improved the patient-clinician relationship. | Not reported. |
| **Prescott and Hanley (2023)** | Prior experience with AI or machine therapy was very limited, with only one participant reporting previous use. | Therapists and trainees were cautious and often skeptical, while recognising selected supportive roles. | Potential to improve access to support, engage people who may otherwise be harder to reach, and provide additional resources alongside therapy. | Weak therapeutic bond, inability to detect nuance and risk, data misuse, inequity, reduced empathy, and harm to vulnerable clients. | Training, initial assessment or triage, and adjunctive support rather than machine-led therapy. | AI was viewed as a complementary tool rather than a replacement for therapists or human clinical judgement. | AI was considered for training, triage, and practice support rather than replacement of therapists. | Participants strongly doubted that AI could form the emotional bond central to therapeutic alliance. | Professional standards, data-governance safeguards, safety requirements, and protection against two-tiered care. |
| **Sharif et al. (2025)** | Awareness was high, clinical use was limited, and role-specific preparedness was low. | Perceptions of AI’s impact were positive. | Participants positively rated AI’s potential impact on mental healthcare. | Low preparedness for role-specific AI integration. | Not reported. | Role-specific preparedness was low. | Professional background and AI awareness were associated with perceptions and preparedness. | Not reported. | Targeted education to improve AI literacy and preparedness and ethical implementation. |
| **Shetty and Rao (2026)** | Direct AI experience was limited. | Professionals were cautiously optimistic; trust was conditional. | Efficiency, reduced workload, triage, large-scale screening, early detection, access, and potentially reduced stigma. | False negatives and positives, unsafe assessment of high-risk presentations, privacy, consent, bias, breaches, opacity, and unclear accountability. | Checklists, structured assessment, risk flagging, triage, and community screening; not independent diagnosis or treatment. | Workflow integration, professional endorsement, and clinician oversight. | AI was not considered suitable for independent diagnosis or treatment and could not replace clinician judgement. | AI could not reproduce empathy, contextual and non-verbal interpretation, or therapeutic relationships. | Empirical validation, transparency, privacy safeguards, training, and governance. |
| **Stroud et al. (2025)** | Not reported. | Clinicians were optimistic about long-term potential, but cautious about near-term clinical risks. | Optimised medication efficacy, reduced administration, shared decision-making, and increased access. | Misguided judgement, added clinical burden, patient harm, and legal liability. | Evaluation, diagnosis, treatment support, and administrative assistance. | Specialist involvement and safety evaluation. | AI should inform shared decisions without displacing specialist judgement or accountability. | Not reported. | Clear responsibility and legal guidance. |
| **Tuncer and Cetinkaya Duman (2025)** | Not reported. | Psychiatric nurses expressed cautious and sometimes negative views of current direct-care applications. | Care-plan support, patient monitoring, administrative assistance, and possible future psychosocial-care support. | Inability to meet emotional needs, patient-safety concerns, integration barriers, uncertainty, and reduced face-to-face care. | Indirect and administrative care, care planning, and monitoring were more acceptable than direct emotional care. | Careful integration into nursing education and care. | AI was more acceptable for indirect and administrative care than replacement of nurses. | AI was considered unable to meet emotional needs; patients’ preference for humans and the importance of face-to-face care were emphasised. | Training, safety safeguards, and preservation of human-centred practice. |
| **Wagner and Schwind (2025)** | Participants varied in their self-reported level of technical affinity, with some describing themselves as not technically inclined. | Attitudes were more positive when AI was perceived as useful. | Potential to support clinical decision-making and treatment through more personalised, responsive, and predictive care. | Fear of professional replacement and limited understanding of AI technologies. | Diagnosis, personalised treatment planning, empathic support, and relapse prediction. | Limited technical familiarity identified as a potential barrier to adoption. | Fear of professional replacement was reported; the human qualities of psychotherapists were emphasised as irreplaceable. | Empathic support was assessed as a possible application. | Education was identified as important for addressing knowledge gaps, fears, and uncertainty. |
| **Zhang et al. (2023)** | Adoption was slow and limited knowledge was reported. | Some resistance was evident, although participants saw constructive potential when implementation was supported. | More efficient, reliable, and higher-quality care and improved capacity to discuss patients’ AI use. | Low confidence, limited adoption, lack of organisational readiness, and insufficient knowledge. | Clinical decision support, suicide-risk prediction, and supporting clinicians’ discussions with clients about AI use. | Practice-change support, self-efficacy, organisational readiness, funding, collaboration, and system-level change. | Humanistic and compassionate care should remain central. | Not reported. | Education and governance. |
